# Long-Term Organic Fertilization Enhances Soil Fertility and Reshapes Microbial Community Structure with Decreasing Effects Across Soil Depth

**DOI:** 10.3390/microorganisms14010250

**Published:** 2026-01-21

**Authors:** Suyao Li, Yulin Li, Xu Yan, Zhengyang Gu, Dong Xue, Kaihua Wang, Yuting Yang, Min Lv, Yujie Han, Jinbiao Li, Yanyan Lv, Anyong Hu

**Affiliations:** 1 School of Geographical Science, Nantong University, Nantong 226019, China; 19517128836@163.com (S.L.); yulinli922@sina.com (Y.L.); yanxu20050129@sina.com (X.Y.); gzy13815927234@163.com (Z.G.); ytyang@ntu.edu.cn (Y.Y.); lm2018@ntu.edu.cn (M.L.); hanyj@ntu.edu.cn (Y.H.); jbli@ntu.edu.cn (J.L.); 2 Jiangsu Yanjiang Institute of Agricultural Sciences, Nantong 226541, China; xuedongjsrg@jaas.ac.cn (D.X.); 19942003@jaas.ac.cn (K.W.)

**Keywords:** long-term fertilization, soil profile, microbial co-occurrence network, nutrient cycling, functional gene abundance

## Abstract

Sustaining agricultural productivity and soil health under intensive cultivation requires a comprehensive understanding of fertilization effects, particularly on deeper soil layers, which has received limited attention compared to surface soils. This study investigated how different fertilization regimes (inorganic, organic, and combined organic–inorganic fertilizers) influence soil physicochemical properties, microbial diversity, community structure, and functional gene abundances at three soil depths (0–20 cm, 20–40 cm, and 40–60 cm) in a 40-year fertilization experiment. Organic fertilization significantly improved topsoil fertility indicators such as soil organic matter (56.6–109.2%), total nitrogen (66.7–122.0%), total phosphorus (198.6–413.2%), and available phosphorus (984.8–1622.1%) and potassium (35.3–438.1%). Compared with the unfertilized control and nitrogen-only treatment, rice yield increased by 97.1–130.5% under NPK and sole organic fertilization, and further increased by 184.1–255.9% under combined organic–inorganic fertilization. However, fertilization effects diminished with soil depth due to limited nutrient mobility. Microbial diversity significantly decreased with depth and was minimally influenced by fertilization treatments. Microbial community structure varied notably among fertilization treatments at the surface layer, mainly driven by soil nutrients, whereas soil depth had a dominant effect on microbial community structure and compositions. Co-occurrence networks showed the highest complexity in surface soil microbial communities, which declined with soil depth, reflecting potential synergistic and mutualistic relationships in topsoil and the adaptation of microbial communities to nutrient-limited conditions in subsoil. Microbial functional gene analyses highlighted clear depth-dependent distributions, with surface layers enriched in decomposition-related genes, while deeper layers favored anaerobic processes. Overall, long-term fertilization exerted strong depth-dependent effects on soil fertility, microbial community structure, and functional potential in paddy soils.

## 1. Introduction

Soil fertility is a fundamental determinant of agricultural productivity and ecosystem sustainability, particularly under intensive cultivation systems where nutrient inputs are continuously applied. Historically, the use of chemical fertilizers has driven substantial yield gains through rapid supply of nitrogen (N), phosphorus (P), and potassium (K) [1]. However, long-term reliance on inorganic fertilizers has also been associated with declines in soil organic matter (SOM), disruption of soil structure, decreased nutrient use efficiency, and secondary issues such as acidification and nutrient leaching [2]. In contrast, organic amendments, such as composted manure or crop residues, can improve soil aggregation, increase water-holding capacity, and provide a slow-release nutrient source that fosters microbial activity and ecosystem resilience [3]. Accordingly, integrated nutrient management, which combines inorganic fertilizers with organic inputs, has been widely recognized as an effective strategy to balance availability of mineral nutrients while sustaining soil fertility, crop productivity, and soil health [4]. However, increasing evidence suggests that soil physicochemical properties vary markedly with depth, microbial community composition, and functional potential, and also exhibit pronounced vertical heterogeneity [5]. Despite this, most studies related to soil nutrient cycling and biochemical processes have focused primarily on surface soils, leaving deeper layers poorly understood.

Soil microorganisms are the primary drivers of soil fertility and ecosystem functioning, as they drive the biogeochemical cycling of carbon, nitrogen, and phosphorus. Through the production of extracellular enzymes, microbial communities decompose complex organic polymers, catalyze the mineralization of nitrogen and phosphorus, and mediate redox-sensitive transformations that regulate carbon turnover and nutrient availability to plants [6]. The taxonomic and functional composition of soil microbial communities, often profiled via α-diversity metrics and gene-based functional assays, directly shapes rates of decomposition, nitrification, denitrification, and phosphorus solubilization. Chemical fertilizers, while capable of rapidly replenishing soil nutrients, have been shown to reduce soil microbial diversity and disrupt community structure when applied exclusively over extended periods [7]. In particular, excessive nitrogen inputs have been reported to significantly decrease bacterial α-diversity [8,9]. Concurrently, prolonged use of chemical fertilizers alters the distribution of microbial functional genes, and suppresses the activity of key enzymes involved in carbon and nitrogen cycling [10,11,12]. Such microbial community shifts may lead to decreased nutrient-use efficiency, ultimately affecting crop yield stability. However, the combined organic–inorganic fertilization significantly improves the transformation efficiency of soil carbon, nitrogen, and phosphorus by regulating microbial community functions. Organic amendments promote the proliferation of functional microbial groups, such as cellulolytic and nitrogen-fixing bacteria, thereby accelerating organic matter mineralization [13,14]. Simultaneously, combining inorganic fertilizers with organic fertilizers maintains more balanced microbial metabolic activity, which in turn enhances nutrient-use efficiency and supports soil fertility and crop productivity [15].

Soil is a highly heterogeneous ecosystem that exhibits pronounced vertical stratification in its physical, chemical, and biological properties [16,17,18]. With increasing soil depth, gradients in nutrient availability, oxygen concentration, moisture content, and root exudates strongly influence microbial community composition, structure and nutrient transformation processes [5,19]. In the topsoil, where root exudates and organic matter inputs are abundant, microbial biomass and diversity typically reach their maximum and are dominated by copiotrophic microorganisms [20,21]. This layer is also highly sensitive to fertilization management, with long-term mineral fertilizer application often leading to reduced microbial diversity and altering the composition of key functional microbial groups [22,23]. In contrast, microbial communities in subsoil layers are primarily regulated by limited resource availability, distinct redox environments, and specialized ecological strategies. Lower soil bacterial diversity is commonly observed in subsoil compared with topsoil, reflecting a depth-dependent response from microbial communities to fertilization disturbances [24,25,26]. Previous studies have further shown that long-term combined application of organic and inorganic fertilizers can distinctly reshape microbial communities along soil profiles, enriching key functional taxa such as Nitrososphaera, Nitrospira, and members of Acidobacteria in topsoil, as well as *Subdivision 3 genera incertae sedis*, *Leptolinea*, and *Bellilinea* in subsoil layers [27].

Our previous study under upland conditions, focusing on faba bean at maturity, revealed pronounced effects of long-term fertilization on soil fertility, microbial communities, and crop yield [28]. However, it remains unknown how these responses differ in paddy soils with rice, where flooded conditions fundamentally alter nutrient cycling and microbial processes through changes in soil redox status. Despite growing recognition of fertilization impacts on farmland ecosystems, especially to soil surface layers, systematic investigations into how inorganic and organic fertilization distinctly modulate soil chemical properties and microbial communities across different soil depths remain limited. Therefore, the aim of this study was to investigate the depth-dependent effects of long-term fertilization on soil fertility and microbial community dynamics based on a unique 40-year long-term field experiment. Specifically, this study aims to (1) characterize the depth-dependent (0–20 cm, 20–40 cm, and 40–60 cm) responses of soil chemical properties to various fertilization regimes (inorganic, organic, and their combinations), (2) elucidate the interactive effects of fertilization practices and soil depth on microbial community diversity, composition, and structure, and (3) reveal how fertilization practices and soil depth shape microbial co-occurrence network topology (complexity, connectivity, and modularity) and microbial functional genes involved in carbon, nitrogen, and phosphorus cycling. This research will improve understanding of depth-dependent microbial responses to fertilization and provide theoretical support for optimizing integrated nutrient management strategies that sustain soil health, enhance nutrient-use efficiency, and ultimately maintain crop productivity and ecosystem stability under intensive cultivation.

## 2. Materials and Methods

### 2.1. Experimental Site

The field experiment was conducted at the long-term experimental site of the Jiangsu Yanjiang Institute of Agricultural Sciences, situated in Xueyao Town, Rugao City, Jiangsu Province, China (120°37′ E, 32°07′ N). This area experiences a subtropical monsoon climate, with an average annual temperature of 16.2 °C and average annual precipitation of approximately 1250 mm. The soil at the site is classified as fluvo-aquic, developed from sediments of the Yangtze River, and characterized by a sandy loam texture. The long-term trial was initiated in 1979. At the beginning of the experiment, the surface soil (0–15 cm) had the following properties: soil organic matter (SOM) content of 14.4 g kg^−1^, alkali-hydrolyzable nitrogen of 133 mg kg^−1^, available phosphorus (AP) of 29.0 mg kg^−1^, available potassium (AK) of 64.0 mg kg^−1^, and a pH of 7.86.

### 2.2. Experimental Design and Soil Sample Collection

A continuous 3-year crop rotation system has been implemented at the site since June 1979, comprising six sequential crops: rice (*Oryza sativa* L.), faba bean (*Vicia faba* L.), maize (*Zea mays* L.), barley (*Hordeum vulgare* L.), cotton (*Gossypium* spp.), and wheat (*Triticum aestivum* L.). Starting from June 2022, after the wheat harvest, soybean (*Glycine max* (L.) Merr.) was planted, and the crop rotation cycle was modified to: wheat–soybean–faba bean–maize–barley–rice. The long-term experiment includes six fertilization treatments: no fertilizer input (CK), nitrogen fertilizer alone (N), a combination of nitrogen, phosphorus, and potassium fertilizers (NPK), organic manure alone (M), nitrogen plus manure (MN), and the combined application of NPK and manure (MNPK). Each plot measured 16.8 m^2^, arranged in a completely randomized block design with three replicates. Faba bean and soybean received nitrogen at a rate of 45 kg N hm^−2^ (as urea), while the other four crops were fertilized with 225 kg N hm^−2^. All six crops were supplied with 24.56 kg P hm^−2^ (as calcium superphosphate) and 139.00 kg K hm^−2^ (as potassium chloride). Urea, calcium superphosphate, and potassium chloride are the predominant mineral fertilizers used in agricultural fields under typical management practices in China. For treatments involving organic inputs (M, MN, and MNPK), pig manure was applied at a rate of 18 t hm^−2^. The pig manure had a pH of 7.6 and contained 312.2 g kg^−1^ of soil organic matter, 5.7 g kg^−1^ total nitrogen, 3.0 g kg^−1^ phosphorus, and 3.6 g kg^−1^ potassium. All phosphorus, potassium, and organic fertilizers were incorporated as basal applications prior to sowing. Nitrogen was top-dressed for faba bean, while for the remaining crops, it was applied in split doses: 40% as basal fertilizer and 60% as top dressing during the growing season. Aboveground plant biomass was manually harvested at crop maturity and was not returned to the field.

Bulk soil samples were collected on 30 October 2024, approximately one week before the rice harvest. From each plot, five soil cores were taken at depths of 0–20 cm, 20–40 cm, and 40–60 cm using a hollow auger and thoroughly homogenized to form a composite sample. The samples were placed into sterile zip-lock plastic bags, transported in an ice-packed cooler box, and promptly delivered to the laboratory. Upon arrival, fresh samples were manually broken up and the fine roots and plant litter were carefully removed by hand as thoroughly as possible. A portion of the fresh soil was immediately used for determining soil moisture content and extracting inorganic nitrogen. Approximately 50 g of each fresh sample was stored at −80 °C for subsequent DNA extraction. The remaining soil was air-dried in the shade and ground to pass through 0.85 mm and 0.149 mm sieves for further analyses.

### 2.3. Soil Properties and Rice Yield Determinations

Soil chemical properties, including pH, electrical conductivity (EC), soil organic matter (SOM), water-soluble organic carbon (WSOC), total nitrogen (TN), total phosphorus (TP), ammonium nitrogen (NH_4_^+^-N), nitrate nitrogen (NO_3_^−^-N), available phosphorus (AP), available potassium (AK), and microbial biomass carbon (MBC), were determined using standard methods described previously, with minor modifications. Soil pH and EC were measured in soil-to-water suspensions (1:2.5 and 1:5, w:v, respectively), and soil organic matter was determined using the potassium dichromate oxidation method [29]. WSOC was extracted with deionized water and quantified using a total organic carbon analyzer, as described by Li et al. [30]. Total nitrogen was measured by high-temperature combustion using an elemental analyzer [31]. Total phosphorus was determined by the acid-soluble molybdenum–antimony colorimetric method [32]. Inorganic nitrogen (NH_4_^+^-N and NO_3_^−^-N) was extracted with 1 M KCl and analyzed using a continuous flow analyzer following standard methods [33]. Available phosphorus was extracted with 0.5 M NaHCO_3_ and quantified colorimetrically according to Olsen et al. [34], while available potassium was extracted with 1 M ammonium acetate and measured using flame photometry [35]. Microbial biomass carbon (MBC) was determined using the chloroform fumigation–extraction method as described by Vance et al. [36], with a conversion factor (kEC) of 0.38.

At the rice maturity stage, all aboveground plant material in each plot was harvested manually and naturally air-dried. Grain yield was determined by manually threshing the dried plants, followed by weighing the grains to obtain yield per plot.

### 2.4. Soil DNA Extraction and High-Throughput Sequencing

Soil DNA was extracted from 0.5 g of fresh soil using the FastDNA Spin Kit (MP Biomedicals, Santa Ana, CA, USA) according to the manufacturer’s instructions. DNA quality and concentration were assessed by 1% agarose gel electrophoresis and NanoDrop 2000 spectrophotometry (Thermo Scientific, Wilmington, DE, USA). The procedures for soil DNA extraction, PCR amplification, library preparation, and high-throughput sequencing generally followed our previous study [28]. Briefly, the V4–V5 hypervariable region of the bacterial 16S rRNA gene was amplified using primers 515F and 907R. PCR products from triplicate reactions were pooled, purified, quantified, and normalized prior to paired-end sequencing on an Illumina MiSeq PE300 platform (Illumina, San Diego, CA, USA) by Majorbio Bio-Pharm Technology Co., Ltd. (Shanghai, China). Raw sequence data have been deposited in the NCBI Sequence Read Archive under accession number PRJNA1304740.

### 2.5. Amplicon Sequence Processing and Analysis

Following demultiplexing, raw reads were trimmed and filtered for quality using fastp (v0.19.6) [37] and then merged with FLASH (v1.2.7) [38]. High-quality merged reads were denoised via the DADA2 [39] plugin in QIIME 2 (v2020.2) [40], yielding single-nucleotide-resolution amplicon sequence variants (ASVs) after chimera removal. To standardize sampling effort, bacterial read counts were rarefied to 27,657 sequences per sample (mean Good’s coverage: 98.90%). ASV taxonomic identities were assigned in QIIME 2 using the built-in Naive Bayes classifier against the SILVA 16S rRNA reference database (v138).

### 2.6. Functional Gene Screening

The Tax4Fun package was used to obtain the abundance of relevant genes involved in the carbon, nitrogen, and phosphorus cycles in soil based on 16S rRNA gene sequencing data [41]. The functional annotations in Tax4Fun2 are based on the Kyoto Encyclopedia of Genes and Genomes (KEGG) database, and all predicted functions are expressed as relative abundances. In accordance with previously published studies [42,43,44,45], 395 KEGG functional pathways were identified and subsequently grouped into 22 metabolic categories related to carbon (C), nitrogen (N), and phosphorus (P) cycling. Detailed information on these functional categories is presented in Table S4.

### 2.7. Statistical Analysis

Statistical analyses of data were carried out using SPSS 26.0 software. Statistical significance at the same soil layer was determined by one-way analysis of variance (ANOVA) followed by Duncan’s test with *p* < 0.05. Two-way ANOVAs were performed to examine the interactive effects of different fertilization treatments and soil depth on soil chemical properties, microbial α-diversity, microbial community composition, and the abundances of microbial functional genes involved in carbon, nitrogen, and phosphorus cycling. Least significant difference (LSD) tests were used for multiple comparisons among treatment means. The test of between-subjects effects was applied to specifically evaluate the interaction between fertilization treatments and soil depth, with statistical significance set at *p* < 0.05. Principle coordinate analysis (PCoA) and redundancy analysis (RDA) were conducted using a ‘microeco’ package [46] in R 4.4.3 (R Core Team 2025) based on Bray–Curtis distances. The linear discriminant analysis (LDA) effect size (LEfSe) approach [47] was applied to determine the differences in microbial communities between treatments under different soil depths. Co-occurrence networks of bacteria were constructed by calculating Spearman correlations among ASVs from different soil layers (18 samples per layer). ASVs with a mean relative abundance >0.01% and detected in more than 9 samples were used for network construction. Correlations with a Spearman’s correlation coefficient |r| > 0.63 and *p* < 0.01 were kept in the network. The correlation network was visualized using Gephi v0.10.1 software [48].

## 3. Results

### 3.1. Effects of Long-Term Fertilization Practices on Soil Properties and Rice Yield

In the topsoil layer (0–20 cm, FL), the organic fertilizer-added treatments (M, MN, and MNPK) significantly improved multiple soil fertility indicators SOM, WSOC, TN, TP, AP, and MBC, which were significantly higher compared to unfertilized (CK) and solely chemically fertilized (N and NPK) treatments (Table 1). Correspondingly, rice yield was markedly enhanced under organic-involved treatments, with the highest yield observed in MN, followed by MNPK and M/NPK, all of which were significantly higher than in CK (2739.46 kg ha^−1^) and N treatments. In the subsoil layers (20–40 cm, SL; 40–60 cm, TL), fertilization effects on most soil fertility parameters were less pronounced. However, Soil EC showed a consistent trend across all three soil layers. CK and N treatments maintained the lowest EC values, while NPK and M treatments moderately increased EC, with no significant difference between them. The highest EC values were observed under MN and MNPK treatments. The two-way ANOVA revealed significant interactions between fertilization treatments and soil depth for key soil properties including SOM, WSOC, TN, TP, AP, AK, and MBC (*p* < 0.001).

### 3.2. Synergistic Effects of Fertilization and Depth on Soil Microbial Diversity and Community Structure

Soil microbial alpha diversity, as indicated by Chao1, Shannon, and Simpson, and phylogenetic diversity (PD) indices varied significantly with soil depth but showed limited responses to fertilization treatments (Table 2). All alpha diversity indices differed markedly among soil depths, with highly significant effects observed for depth (*p* < 0.001). However, no significant differences were detected among fertilization treatments (CK, N, NPK, M, MN, and MNPK) for any index (*p* > 0.05). Furthermore, there were no significant interactions (*p* > 0.05) for any alpha diversity index between fertilization treatment and soil depth.

**Table 1:** Soil properties and rice grain yields under different fertilization treatments and soil depths.

Treatments × Depth	pH	EC (μs cm^−1^)	SOM (g kg^−1^)	WSOC (mg kg^−1^)	TN (g kg^−1^)	TP (g kg^−1^)	NO_3_^−^-N (mg kg^−1^)	NH_4_^+^-N (mg kg^−1^)	AP (mg kg^−1^)	AK (mg kg^−1^)	MBC (mg kg^−1^)	GY (kg ha^−1^)
CK_FL	7.81 ± 0.52 a	182.9 ± 17.2 b	15.2 ± 0.9 b	49.0 ± 3.1 b	0.41 ± 0.00 b	0.53 ± 0.10 c	3.20 ± 1.07 a	25.6 ± 3.7 bc	7.3 ± 0.7 b	92.5 ± 8.3 d	145.9 ± 18.5 c	2739.5 ± 663.2 c
N_FL	8.03 ± 0.19 a	172.8 ± 8.6 b	15.6 ± 1.9 b	52.3 ± 5.3 b	0.43 ± 0.02 b	0.63 ± 0.01 c	2.71 ± 0.36 a	23.8 ± 4.5 c	6.9 ± 1.6 b	84.9 ± 9.4 d	217.8 ± 20.0 bc	2342.4 ± 587.5 c
NPK_FL	8.05 ± 0.12 a	293.7 ± 103.2 ab	18.1 ± 1.1 b	59.1 ± 4.5 b	0.51 ± 0.02 b	0.70 ± 0.06 c	3.02 ± 1.04 a	34.5 ± 2.9 a	9.1 ± 2.0 b	135.9 ± 10.4 cd	215.0 ± 13.6 bc	5399.5 ± 537.1 b
M_FL	8.02 ± 0.11 a	298.3 ± 106.7 ab	31.3 ± 2.6 a	109.7 ± 5.3 a	0.87 ± 0.09 a	2.17 ± 0.31 b	1.87 ± 1.40 a	32.6 ± 3.0 ab	111.6 ± 9.7 a	331.2 ± 73.4 b	284.0 ± 53.5 ab	5399.5 ± 299.8 b
MN_FL	8.01 ± 0.08 a	488.0 ± 203.0 a	28.3 ± 0.3 a	109.1 ± 6.1 a	0.85 ± 0.04 a	2.09 ± 0.34 b	1.89 ± 1.33 a	28.5 ± 4.6 abc	99.2 ± 6.4 a	183.9 ± 9.2 c	359.0 ± 86.5 a	8337.5 ± 412.6 a
MNPK_FL	7.95 ± 0.11 a	504.3 ± 156.1 a	31.7 ± 2.8 a	109.8 ± 9.7 a	0.91 ± 0.13 a	2.72 ± 0.16 a	3.00 ± 0.57 a	27.9 ± 6.4 abc	119.0 ± 24.1 a	456.8 ± 91.0 a	325.0 ± 50.2 a	7781.7 ± 181.9 a
CK_SL	7.97 ± 0.27 a	202.4 ± 61.0 b	9.8 ± 0.8 a	29.5 ± 1.8 b	0.27 ± 0.01 c	0.51 ± 0.01 b	2.64 ± 0.99 a	20.6 ± 3.6 a	6.8 ± 0.8 a	84.7 ± 3.4 ab	59.2 ± 4.5 ab	—
N_SL	8.10 ± 0.17 a	170.5 ± 11.5 b	11.5 ± 1.7 a	36.9 ± 3.8 a	0.30 ± 0.04 abc	0.55 ± 0.04 b	2.60 ± 0.30 a	23.2 ± 2.1 a	9.3 ± 3.6 a	80.7 ± 9.0 abc	40.9 ± 8.8 b	—
NPK_SL	8.07 ± 0.09 a	304.3 ± 144.1 ab	10.7 ± 0.4 a	31.4 ± 2.7 b	0.29 ± 0.01 bc	0.59 ± 0.01 b	2.48 ± 0.21 a	22.9 ± 4.9 a	8.9 ± 0.6 a	77.1 ± 10.9 bc	58.6 ± 28.8 ab	—
M_SL	8.15 ± 0.14 a	329.6 ± 118.4 ab	11.7 ± 1.0 a	37.6 ± 2.5 a	0.31 ± 0.01 ab	0.97 ± 0.46 a	2.47 ± 0.20 a	24.6 ± 2.5 a	9.7 ± 2.9 a	80.0 ± 9.5 bc	42.1 ± 9.1 b	—
MN_SL	8.10 ± 0.04 a	506.8 ± 234.9 a	10.7 ± 1.1 a	40.3 ± 0.7 a	0.30 ± 0.02 abc	0.69 ± 0.12 ab	2.15 ± 0.75 a	24.8 ± 4.5 a	9.8 ± 2.9 a	66.5 ± 10.2 c	74.6 ± 17.8 a	—
MNPK_SL	8.00 ± 0.10 a	529.2 ± 178.0 a	10.8 ± 0.6 a	37.8 ± 0.6 a	0.33 ± 0.01 a	0.73 ± 0.04 ab	2.55 ± 0.36 a	22.6 ± 2.7 a	10.2 ± 3.6 a	96.7 ± 6.2 a	50.8 ± 15.5 ab	—
CK_TL	7.97 ± 0.17 a	225.6 ± 83.0 b	7.8 ± 1.3 a	25.0 ± 2.4 b	0.21 ± 0.03 a	0.55 ± 0.01 b	1.93 ± 0.23 a	20.8 ± 5.1 b	9.4 ± 0.9 a	65.9 ± 9.2 a	44.0 ± 7.1 a	—
N_TL	8.17 ± 0.19 a	182.9 ± 23.0 b	8.2 ± 1.8 a	29.2 ± 1.9 ab	0.20 ± 0.08 a	0.55 ± 0.03 b	2.08 ± 0.18 a	23.3 ± 3.7 ab	7.4 ± 0.6 a	63.5 ± 10.8 a	34.8 ± 12.2 a	—
NPK_TL	8.16 ± 0.20 a	304.1 ± 166.8 ab	7.7 ± 2.1 a	24.4 ± 2.5 b	0.20 ± 0.04 a	0.57 ± 0.02 ab	2.86 ± 1.22 a	29.8 ± 4.0 a	10.6 ± 0.8 a	65.1 ± 15.7 a	12.3 ± 1.0 b	—
M_TL	8.21 ± 0.04 a	348.2 ± 79.1 ab	7.9 ± 0.5 a	26.5 ± 1.2 ab	0.19 ± 0.02 a	0.61 ± 0.05 ab	1.96 ± 0.40 a	27.2 ± 3.2 ab	8.4 ± 5.3 a	57.9 ± 10.6 a	25.3 ± 8.5 ab	—
MN_TL	8.13 ± 0.08 a	525.7 ± 277.8 a	8.2 ± 1.5 a	30.8 ± 5.6 a	0.21 ± 0.05 a	0.69 ± 0.07 a	2.03 ± 0.72 a	26.9 ± 4.9 ab	12.2 ± 2.6 a	56.2 ± 8.8 a	40.3 ± 16.0 a	—
MNPK_TL	8.10 ± 0.02 a	564.1 ± 138.2 a	8.1 ± 0.6 a	27.2 ± 1.6 ab	0.22 ± 0.02 a	0.65 ± 0.13 ab	2.61 ± 0.13 a	22.8 ± 3.6 ab	9.7 ± 4.1 a	64.5 ± 3.2 a	14.2 ± 8.1 b	—
Treatments	ns	***	***	***	***	***	ns	**	***	***	***	***
Depth	ns	ns	***	***	***	***	ns	**	***	***	***	—
Treatments × Depth	ns	ns	***	***	***	***	ns	ns	***	***	***	—

Data represent means ± SD (n = 3). Means in each column not sharing any lowercase letters at the same soil layer are significantly different (*p* < 0.05, Duncan’s test). ns—not significant; **—*p* < 0.01; ***— *p* < 0.001 (LSD); CK—no fertilization; N—only nitrogen fertilizer; NPK—nitrogen, phosphorus, and potassium fertilizers; M—only organic fertilizer; MN—N + M; MNPK—NPK + M; FL—0–20 cm soil layer; SL—20–40 cm soil layer; TL—40–60 cm soil layer; EC—electrical conductivity; SOM—soil organic matter; WSOC—water-soluble organic carbon; TN—total nitrogen; TP—total phosphorus; AP—available phosphorus; AK—available potassium; MBC—microbial biomass carbon; GY—grain yield.

**Table 2:** Alpha diversity of soil microbial communities under different fertilization treatments and soil depths.

Treatments × Depth	Chao1	Shannon	Simpson	Phylogenetic Diversity
CK_FL	2753 ± 134 a	7.08 ± 0.08 ab	0.0016 ± 0.0002 bc	378.1 ± 9.8 a
N_FL	2758 ± 196 a	7.06 ± 0.05 ab	0.0017 ± 0.0002 bc	378.0 ± 9.4 a
NPK_FL	2963 ± 17 a	7.14 ± 0.02 a	0.0016 ± 0.0001 c	400.8 ± 6.8 a
M_FL	2858 ± 182 a	6.99 ± 0.07 b	0.0023 ± 0.0003 a	390.0 ± 17.3 a
MN_FL	2988 ± 417 a	7.14 ± 0.07 a	0.0016 ± 0.0001 bc	403.2 ± 46.6 a
MNPK_FL	3019 ± 241 a	7.08 ± 0.12 ab	0.0021 ± 0.0005 ab	404.5 ± 23.7 a
CK_SL	2347 ± 117 a	6.78 ± 0.04 a	0.0026 ± 0.0003 a	314.5 ± 8.7 a
N_SL	2092 ± 671 a	6.69 ± 0.27 a	0.0027 ± 0.0006 a	290.5 ± 80.7 a
NPK_SL	2145 ± 162 a	6.66 ± 0.08 a	0.0029 ± 0.0003 a	294.3 ± 28.1 a
M_SL	2533 ± 481 a	6.86 ± 0.23 a	0.0025 ± 0.0009 a	345.2 ± 59.1 a
MN_SL	2339 ± 620 a	6.72 ± 0.29 a	0.0033 ± 0.0010 a	323.1 ± 67.7 a
MNPK_SL	2381 ± 547 a	6.69 ± 0.19 a	0.0032 ± 0.0004 a	318.4 ± 64.4 a
CK_TL	1965 ± 29 a	6.63 ± 0.05 a	0.0033 ± 0.0003 a	267.7 ± 3.2 a
N_TL	2264 ± 240 a	6.83 ± 0.12 a	0.0025 ± 0.0005 a	317.0 ± 46.7 a
NPK_TL	1867 ± 114 a	6.61 ± 0.05 a	0.0036 ± 0.0000 a	261.1 ± 11.7 a
M_TL	2192 ± 407 a	6.72 ± 0.16 a	0.0033 ± 0.0005 a	306.8 ± 39.8 a
MN_TL	2329 ± 388 a	6.81 ± 0.23 a	0.0027 ± 0.0011 a	311.6 ± 41.4 a
MNPK_TL	2329 ± 42 a	6.72 ± 0.11 a	0.0033 ± 0.0006 a	311.7 ± 16.2 a
Treatment	ns	ns	ns	ns
Depth	***	***	***	***
Treatments × Depth	ns	ns	ns	ns

Data represent means ± SD (n = 3). Means in each column not sharing any lowercase letters at the same soil layer are significantly different (*p* < 0.05, Duncan’s test). ns—not significant; ***—*p* < 0.001 (LSD); CK—no fertilization; N—only nitrogen fertilizer; NPK—nitrogen, phosphorus, and potassium fertilizers; M—only organic fertilizer; MN—N + M; MNPK—NPK + M; FL—0–20 cm soil layer; SL—20–40 cm soil layer; TL—40–60 cm soil layer.

**Figure 1:**
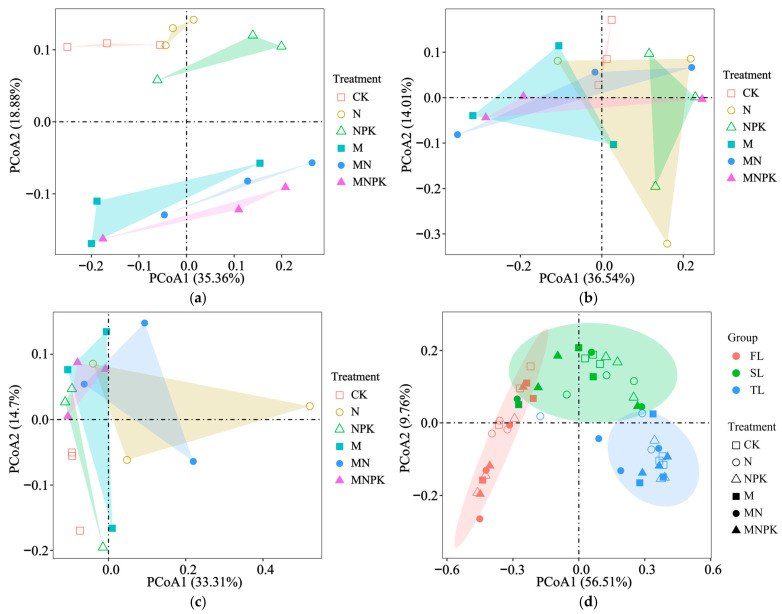
Principal coordinate analysis of microbial community in the first (0–20 cm, (**a**)), second (20–40 cm, (**b**)), and third (40–60 cm, (**c**)) soil layers, as well as across all soil layers combined (**d**), under different fertilization treatments. CK—no fertilization; N—only nitrogen fertilizer; NPK—nitrogen, phosphorus and potassium fertilizers; M—only organic fertilizer; MN—N + M; MNPK—NPK + M. FL—0–20 cm soil layer; SL—20–40 cm soil layer; TL—40–60 cm soil layer.

In the surface soil (Figure 1a), microbial communities under different fertilization regimes were clearly separated. Treatments receiving organic fertilizer (M, MN, and MNPK) clustered tightly together and were significantly separated from inorganic-only (N and NPK) and unfertilized (CK) treatments (*p* < 0.05). In contrast, in the middle (Figure 1b) and deep layers (Figure 1c), the separation among fertilization treatments was less distinct, with substantial overlap among samples (*p* > 0.05). When samples from all soil layers were analyzed together (Figure 1d), microbial community structure was primarily differentiated by soil depth rather than fertilization treatment (*p* < 0.05), indicating that vertical soil stratification results in a stronger influence on overall community structure than fertilization treatments.

**Figure 2:**
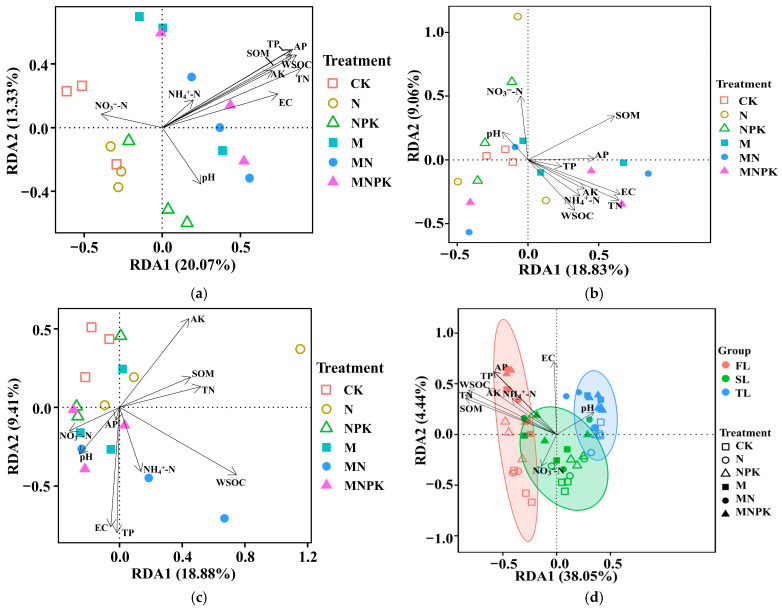
Redundancy analysis of microbial community in the first (0–20 cm, (**a**)), second (20–40 cm, (**b**)), and third (40–60 cm, (**c**)) soil layers, as well as across all soil layers combined (**d**), under different fertilization treatments. CK—no fertilization; N—only nitrogen fertilizer; NPK—nitrogen, phosphorus, and potassium fertilizers; M—only organic fertilizer; MN—N + M; MNPK—NPK + M; EC—electrical conductivity; SOM—soil organic matter; WSOC—water-soluble organic carbon; TN—total nitrogen; TP—total phosphorus; AP—available phosphorus; AK—available potassium.

In the surface soil, microbial communities were strongly structured along nutrient-related gradients, with vectors for TN, WSOC, AP, TP, and SOM, which exerted the strongest influence on microbial community structure, pointing toward organically amended treatments (Figure 2a and Table S1). EC and AK also contributed to community separation, although their effects were weaker. In the middle soil layer, most environmental vectors shortened and converged toward the origin, indicating diminished environmental control and explanatory power (Figure 2b and Table S1). In the deep soil layer, microbial communities exhibited pronounced convergence irrespective of fertilization treatments. Environmental vectors were short and poorly aligned with sample distribution, suggesting that none of the measured soil properties exerted strong control over microbial community structure in deep soil (Figure 2c and Table S1). When all soil layers were analyzed together, surface, middle, and deep soil communities were clearly separated along the RDA1 axis according to soil depth (Figure 2d and Table S1). Nutrient-related variables (TN, SOM, WSOC, AK, TP, and AP) were closely associated with surface soil samples, whereas pH showed a stronger association with deeper layers (Table S1).

### 3.3. Soil Microbial Community Composition and Specific Lefse Biomarkers Under Fertilization–Depth Coupling

**Figure 3:**
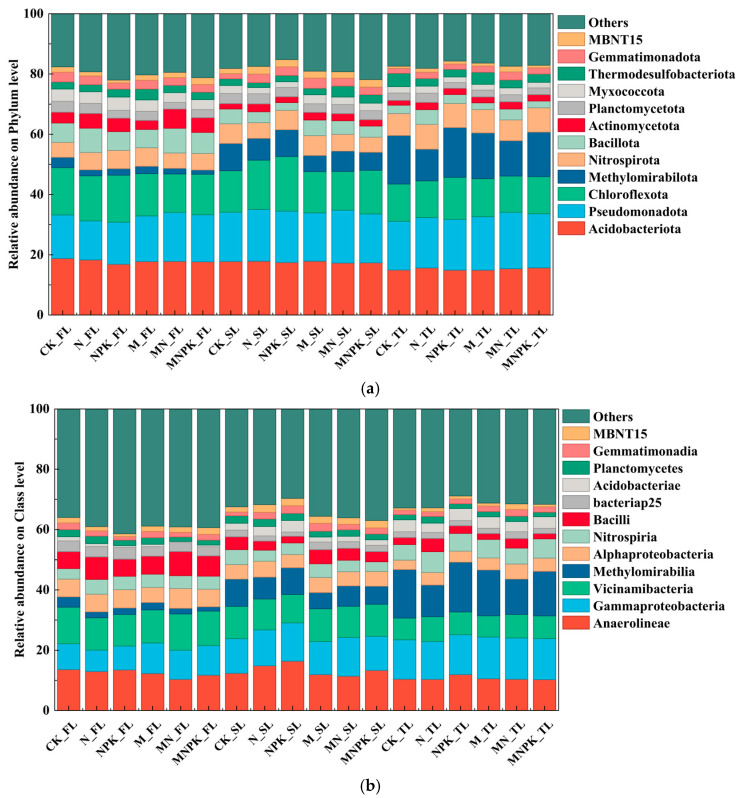
Composition of soil microbial community at the phylum (**a**) and class (**b**) rank under different fertilization treatments and soil depth. CK—no fertilization; N—only nitrogen fertilizer; NPK—nitrogen, phosphorus, and potassium fertilizers; M—only organic fertilizer; MN—N + M; MNPK—NPK + M; FL—0–20 cm soil layer; SL—20–40 cm soil layer; TL—40–60 cm soil layer.

Across all fertilization treatments and soil depths, twelve dominant bacterial phyla were identified, with Acidobacteriota, Pseudomonadota, and Chloroflexota together accounting for nearly half of the total community abundance (Figure 3a). Overall, soil depth emerged as the primary determinant of phylum-level distribution differences. Six phyla, including Acidobacteriota, Chloroflexota, Bacillota, Actinomycetota, Planctomycetota, and Myxococcota exhibited significant and consistent declines from topsoil to deep soil layers (Table S2, *p* < 0.01 or *p* < 0.001). Conversely, Pseudomonadota, Methylomirabilota, Nitrospirota, and MBNT15 showed marked enrichment in deeper soil horizons. Long-term fertilization treatments, such as Chloroflexota and Planctomycetota, had only minor effects at phylum level.

At the class level, twelve dominant taxa collectively characterized the microbial community structure (Figure 3b). Similar to the phylum-level trend, soil depth strongly shaped class-level distributions. Several classes, including Anaerolineae, Vicinamibacteria, Alphaproteobacteria, Bacilli, bacteriap25, Planctomycetes, Gemmatimonadia, MBNT15, and Planctomycetes all exhibited significant declines with depth (Table S3, *p* < 0.01). In contrast, Gammaproteobacteria, Methylomirabilia, Nitrospiria, and Acidobacteriae increased in abundance in deeper soil layers (Table S3, *p* < 0.01 or *p* < 0.001). Fertilization treatments showed negligible effects on most dominant classes, with statistically significant responses detected only for Anaerolineae and Planctomycetes. Together, these phylum-level and class-level results confirm that soil depths rather than fertilization treatments are the principal shapers of soil microbial community composition.

To pinpoint the microbial taxa most strongly associated with fertilization treatments, we integrated the taxonomic distribution from the LEfSe cladograms (Figure S1) with their LDA effect sizes (Figure S2). Using an LDA threshold of >2, we detected 94, 28, and 25 discriminative biomarkers in the surface (0–20 cm), middle (20–40 cm), and deep (40–60 cm) soil layers, respectively. In the surface layer, 23, 14, 10, 4, 22, and 21 biomarkers were uniquely associated with CK, N, NPK, M, MN, and MNPK treatments (Figure S2a). The most significant biomarkers (LDA > 3.0) included p_Myxococcota, o_Ardenticatenales, o_Gemmatales, and f_Gemmataceae in CK, c_Blastocatellia, o_Pyrinomonadales, and f_Pyrinomonadaceae in N, o_Syntrophobacterales, *g_Syntrophobacter*, c_Syntrophobacteria, and f_Syntrophobacteraceae in NPK, f_Desulfuromonadaceae in M, p_Actinomycetota, c_Thermoleophilia, o_Thermoactinomycetales, and f_Thermoactinomycetaceae in MN. In the middle layer, 7, 8, 2, 4, 4, and 3 biomarkers characterized CK, N, NPK, M, MN, and MNPK (Figure S2b). The only microbial taxa exceeding LDA > 3.0 were o_Rokubacteriales, o_Gemmatales, and f_Gemmataceae in CK. In the deep layer, 8, 3, 3, 3, 5, and 3 biomarkers defined CK, N, NPK, M, MN, and MNPK (Figure S2c). CK was marked by enrichment of o_Geobacterales, f_Geobacteraceae, and *g_Sh765B_TzT_35*, whereas NPK uniquely enriched c_Dehalococcoidia (LDA > 3.0).

### 3.4. Depth-Dependent Co-Occurrence Networks: Topsoil Complexity to Deep-Layer Fragmentation

**Figure 4:**
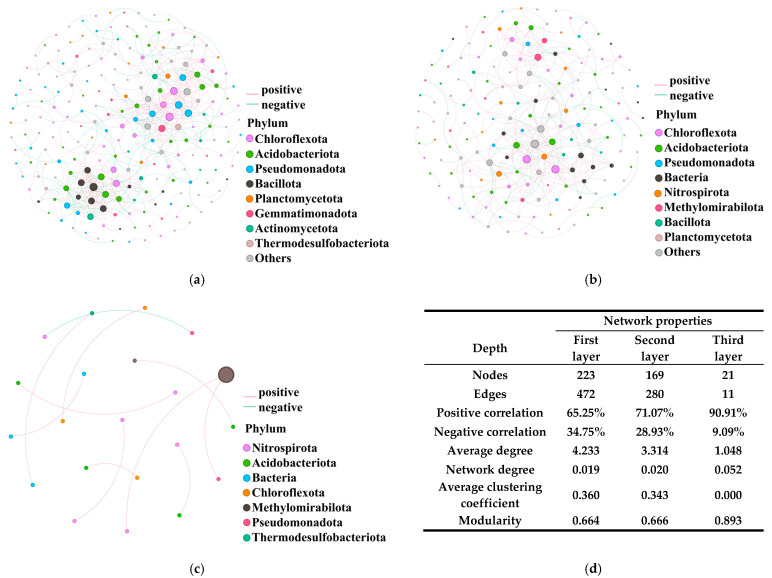
Co-occurrence networks of microbial community at the phylum rank of the first (0–20 cm, (**a**)), second (20–40 cm, (**b**)), and third (40–60 cm, (**c**)) soil layers were constructed using 18 samples (6 fertilization treatments × 3 replicates) obtained from long-term experimental fields by calculating Spearman correlations among ASVs with mean relative abundance > 0.01% and detected in more than 9 samples at each soil layer. (**d**) Basic Network properties of different soil layers in the co-occurrence networks.

To explore the ecological interactions and network stability within soil microbial communities under long-term fertilization, co-occurrence networks were constructed for the surface (0–20 cm), middle (20–40 cm), and deep (40–60 cm) soil layers (Figure 4a–c), with quantitative network properties summarized in Figure 4d. The co-occurrence network of the surface soil displayed the highest complexity and connectivity, characterized by a large number of nodes (223) and edges (472), as well as a high average degree and clustering coefficient. Notably, positive correlations dominated the network (65.25%), suggesting a prevalence of synergistic and potentially cooperative interactions among major bacterial phyla such as Chloroflexota, Acidobacteriota, Pseudomonadota, Bacillota, and Gemmatimonadota. The modularity value (0.664) indicates moderate compartmentalization, suggesting that functional microbial modules are well developed in the topsoil. Network complexity decreased in the middle layer, with the number of nodes and edges reduced to 169 and 280, respectively. The average degree dropped to 3.314, and the clustering coefficient to 0.343. However, the proportion of positive correlations increased to 71.07%, implying an even greater dominance of positive associations, despite reduced network density. The modularity remained stable (0.666), reflecting persistent, albeit less complex, modularity within the microbial community. The observed reduction in network connectivity and complexity suggests that microbial interactions and potential niche differentiation are less pronounced at this depth. In the deep soil, the network was highly simplified, with only 21 nodes and 11 edges. The average degree (1.048) and clustering coefficient (0.000) were markedly lower than in the upper layers. Positive correlations reached 90.91%, while negative interactions were minimal (9.09%) and the modularity increased sharply to 0.893, indicating sparse and highly fragmented microbial associations.

### 3.5. Influence of Soil Depth and Fertilization Practices on Microbial Functional Gene Abundances in Carbon, Nitrogen, and Phosphorus Cycling

In order to directly assess soil biogeochemical functional potential, microbial functional genes involved in C, N, and P cycling were classified according to their metabolic pathways and ecological functions (Table S4). Table 3 shows that the abundances of carbon C–related microbial functional genes were primarily structured by soil depth rather than fertilization treatment. Two-way ANOVAs indicated that soil depth had highly significant effects on all examined functional gene categories, whereas fertilization treatment effects were generally weak or non-significant, and no significant treatment × depth interactions were detected. Across all treatments, genes associated with the degradation of complex organic substrates, including cellulose, hemicellulose, lignin, chitin, starch, and pectin, exhibited a clear decreasing trend with increasing soil depth. These decomposition-related genes were consistently more abundant in the surface soil and declined markedly in deeper layers, reflecting reduced organic substrate availability and microbial activity with depth. In contrast, genes related to carbon fixation and methanogenesis/methane metabolism showed significant increasing abundances in deeper soils. Fertilization treatments exerted only limited influence on C–related functional genes. While minor variations were observed for specific gene categories, these effects were small compared to the pronounced depth-driven differentiation.

Two-way ANOVAs revealed that soil depth had highly significant effects on all nitrogen cycling pathways examined (*p* < 0.001), including nitrification, denitrification, assimilatory and dissimilatory nitrate reduction, nitrogen fixation, anammox, nitrogen mineralization, and nitrogen assimilation (Table 4). In contrast, fertilization effects were generally non-significant or weak, and treatment × depth interactions were largely absent except for nitrogen fixation genes. Across all fertilization treatments, nitrification- and denitrification-related genes exhibited an increasing trend with soil depth, with the highest abundances consistently observed in the deep soil layer (40–60 cm). Similarly, genes involved in dissimilatory nitrate reduction, anammox, and nitrogen mineralization were enriched in deeper layers. In contrast, assimilatory nitrate reduction genes tended to be more abundant in surface soils. Nitrogen fixation genes showed comparatively low abundances overall but displayed significant depth-dependent variation and a detectable interaction between fertilization and soil depth. Despite minor variations among fertilization treatments, nitrogen assimilation genes remained relatively stable across treatments but increased slightly with depth.

Soil depth exerted significant effects on most P cycling pathways, including polyphosphate consolidation, polyphosphate degradation, P uptake and transport systems, inorganic P solubilization, and organic P mineralization (Table 5). In contrast, fertilization treatments had limited or pathway-specific effects and treatment × depth interactions were generally weak or non-significant. Across all fertilization regimes, genes associated with polyphosphate consolidation, P uptake, and transport systems displayed relatively high abundances in surface soils (0–20 cm). With increasing soil depth, these gene abundances tended to decline. In contrast, genes related to polyphosphate degradation and inorganic P solubilization exhibited more pronounced depth-dependent variation, showing higher abundances in subsoil layers. Organic and chemical combined fertilization treatments slightly reduced the abundance of P starvation responses and inorganic P solubilization genes in surface soils. Genes involved in organic P mineralization showed relatively stable abundances across fertilization treatments but decreased progressively with soil depth.

**Table 3:** Carbon cycle-related microbial functional gene abundances under different fertilization treatments and soil depths.

Treatments × Depth	Carbon Fixation	Cellulose Breakdown	Hemicellulose Breakdown	Lignin Breakdown	Chitin Breakdown	Starch Breakdown	Pectin Breakdown	Methanogenesis and Methane Metabolism
CK_FL	1.682 ± 0.054 ab	0.081 ± 0.007 a	0.134 ± 0.014 a	0.021 ± 0.003 abc	0.039 ± 0.002 a	0.108 ± 0.004 ab	0.005 ± 0.001 a	0.534 ± 0.014 a
N_FL	1.687 ± 0.026 ab	0.068 ± 0.009 ab	0.136 ± 0.011 a	0.018 ± 0.003 bc	0.037 ± 0.005 ab	0.113 ± 0.007 a	0.006 ± 0.001 a	0.519 ± 0.007 ab
NPK_FL	1.643 ± 0.018 ab	0.068 ± 0.014 ab	0.133 ± 0.011 a	0.019 ± 0.001 abc	0.035 ± 0.003 ab	0.105 ± 0.004 ab	0.006 ± 0.001 a	0.508 ± 0.005 bc
M_FL	1.712 ± 0.059 a	0.057 ± 0.008 b	0.125 ± 0.009 a	0.017 ± 0.003 c	0.031 ± 0.002 b	0.097 ± 0.009 b	0.005 ± 0.001 a	0.513 ± 0.011 ab
MN_FL	1.614 ± 0.019 b	0.071 ± 0.005 ab	0.147 ± 0.004 a	0.023 ± 0.003 a	0.036 ± 0.003 ab	0.107 ± 0.005 ab	0.005 ± 0.002 a	0.487 ± 0.014 c
MNPK_FL	1.686 ± 0.033 ab	0.073 ± 0.012 ab	0.139 ± 0.019 a	0.023 ± 0.001 ab	0.032 ± 0.002 b	0.100 ± 0.006 b	0.006 ± 0.003 a	0.506 ± 0.015 bc
CK_SL	1.765 ± 0.036 a	0.086 ± 0.021 a	0.147 ± 0.015 a	0.013 ± 0.001 a	0.040 ± 0.003 a	0.099 ± 0.006 ab	0.010 ± 0.004 a	0.544 ± 0.020 a
N_SL	1.779 ± 0.083 a	0.084 ± 0.046 a	0.151 ± 0.056 a	0.011 ± 0.004 a	0.040 ± 0.014 a	0.104 ± 0.005 ab	0.013 ± 0.012 a	0.530 ± 0.030 a
NPK_SL	1.740 ± 0.006 a	0.083 ± 0.040 a	0.161 ± 0.032 a	0.010 ± 0.002 a	0.042 ± 0.005 a	0.107 ± 0.012 a	0.011 ± 0.007 a	0.520 ± 0.010 a
M_SL	1.731 ± 0.062 a	0.062 ± 0.011 a	0.123 ± 0.010 a	0.015 ± 0.006 a	0.031 ± 0.003 a	0.106 ± 0.003 ab	0.004 ± 0.001 a	0.520 ± 0.015 a
MN_SL	1.753 ± 0.071 a	0.059 ± 0.014 a	0.130 ± 0.020 a	0.017 ± 0.006 a	0.037 ± 0.001 a	0.098 ± 0.013 ab	0.005 ± 0.001 a	0.522 ± 0.025 a
MNPK_SL	1.820 ± 0.070 a	0.065 ± 0.010 a	0.125 ± 0.006 a	0.013 ± 0.004 a	0.033 ± 0.003 a	0.089 ± 0.008 b	0.005 ± 0.002 a	0.546 ± 0.025 a
CK_TL	1.883 ± 0.023 a	0.047 ± 0.003 a	0.107 ± 0.003 a	0.009 ± 0.001 a	0.026 ± 0.002 a	0.086 ± 0.006 b	0.003 ± 0.000 a	0.583 ± 0.015 a
N_TL	1.836 ± 0.142 a	0.053 ± 0.019 a	0.118 ± 0.017 a	0.012 ± 0.007 a	0.029 ± 0.005 a	0.106 ± 0.008 a	0.004 ± 0.003 a	0.552 ± 0.024 ab
NPK_TL	1.824 ± 0.036 a	0.050 ± 0.002 a	0.117 ± 0.007 a	0.011 ± 0.002 a	0.030 ± 0.001 a	0.101 ± 0.007 a	0.003 ± 0.001 a	0.564 ± 0.016 a
M_TL	1.835 ± 0.046 a	0.044 ± 0.004 a	0.104 ± 0.014 a	0.012 ± 0.006 a	0.027 ± 0.005 a	0.095 ± 0.002 ab	0.002 ± 0.000 a	0.563 ± 0.003 a
MN_TL	1.812 ± 0.076 a	0.049 ± 0.015 a	0.117 ± 0.014 a	0.017 ± 0.002 a	0.029 ± 0.002 a	0.103 ± 0.010 a	0.004 ± 0.002 a	0.530 ± 0.012 b
MNPK_TL	1.911 ± 0.053 a	0.043 ± 0.007 a	0.105 ± 0.006 a	0.015 ± 0.002 a	0.026 ± 0.000 a	0.101 ± 0.010 a	0.002 ± 0.001 a	0.566 ± 0.019 a
Treatment	ns	ns	ns	*	*	*	ns	**
Depth	***	***	***	***	***	*	**	***
Treatments × Depth	ns	ns	ns	ns	ns	ns	ns	ns

Data represent means ± SD (n = 3). Means in each row not sharing any lowercase letters at the end of each value are significantly different (*p* < 0.05, Duncan’s test). ns—not significant; *—*p* < 0.05; **—*p* < 0.01; ***—*p* < 0.001 (LSD); CK—no fertilization; N—only nitrogen fertilizer; NPK—nitrogen, phosphorus, and potassium fertilizers; M—only organic fertilizer; MN—N + M; MNPK—NPK + M; FL—0–20 cm soil layer; SL—20–40 cm soil layer; TL—40–60 cm soil layer.

**Table 4:** Nitrogen cycle-related microbial functional gene abundances under different fertilization treatments and soil depths.

Treatments × Depth	Nitrification	Denitrification	Assimilatory Nitrate Reduction	Dissimilatory Nitrate Reduction	Nitrogen Fixation	Anammox	Nitrogen Mineralization	Nitrogen Assimilation
CK_FL	0.078 ± 0.018 ab	0.137 ± 0.016 ab	0.323 ± 0.006 ab	0.169 ± 0.009 abc	0.036 ± 0.009 b	0.097 ± 0.021 a	0.055 ± 0.008 a	0.307 ± 0.009 a
N_FL	0.089 ± 0.008 a	0.148 ± 0.006 a	0.315 ± 0.002 b	0.182 ± 0.009 a	0.034 ± 0.002 b	0.093 ± 0.008 ab	0.053 ± 0.003 a	0.308 ± 0.004 a
NPK_FL	0.070 ± 0.007 bc	0.135 ± 0.012 ab	0.327 ± 0.015 ab	0.170 ± 0.009 ab	0.051 ± 0.013 ab	0.080 ± 0.009 ab	0.046 ± 0.004 ab	0.301 ± 0.004 a
M_FL	0.068 ± 0.003 bc	0.131 ± 0.004 ab	0.316 ± 0.005 b	0.175 ± 0.008 ab	0.061 ± 0.017 a	0.075 ± 0.006 b	0.050 ± 0.003 a	0.306 ± 0.009 a
MN_FL	0.059 ± 0.008 c	0.122 ± 0.004 b	0.327 ± 0.009 ab	0.154 ± 0.008 c	0.038 ± 0.005 b	0.072 ± 0.006 b	0.039 ± 0.006 b	0.302 ± 0.007 a
MNPK_FL	0.068 ± 0.005 bc	0.132 ± 0.005 ab	0.333 ± 0.008 a	0.165 ± 0.007 bc	0.046 ± 0.006 ab	0.081 ± 0.004 ab	0.048 ± 0.007 ab	0.306 ± 0.005 a
CK_SL	0.091 ± 0.002 a	0.153 ± 0.004 a	0.290 ± 0.002 a	0.179 ± 0.012 a	0.051 ± 0.007 a	0.108 ± 0.011 a	0.062 ± 0.005 a	0.310 ± 0.003 a
N_SL	0.115 ± 0.023 a	0.172 ± 0.015 a	0.295 ± 0.013 a	0.186 ± 0.010 a	0.043 ± 0.003 a	0.139 ± 0.028 a	0.065 ± 0.010 a	0.318 ± 0.014 a
NPK_SL	0.096 ± 0.010 a	0.165 ± 0.012 a	0.289 ± 0.015 a	0.187 ± 0.006 a	0.050 ± 0.008 a	0.112 ± 0.020 a	0.056 ± 0.004 a	0.317 ± 0.014 a
M_SL	0.099 ± 0.024 a	0.163 ± 0.024 a	0.315 ± 0.019 a	0.189 ± 0.014 a	0.045 ± 0.008 a	0.115 ± 0.028 a	0.060 ± 0.010 a	0.317 ± 0.015 a
MN_SL	0.092 ± 0.021 a	0.157 ± 0.025 a	0.309 ± 0.014 a	0.186 ± 0.020 a	0.054 ± 0.032 a	0.105 ± 0.017 a	0.056 ± 0.008 a	0.325 ± 0.015 a
MNPK_SL	0.097 ± 0.028 a	0.154 ± 0.023 a	0.300 ± 0.016 a	0.172 ± 0.007 a	0.050 ± 0.019 a	0.124 ± 0.047 a	0.065 ± 0.005 a	0.322 ± 0.013 a
CK_TL	0.112 ± 0.018 a	0.188 ± 0.017 a	0.289 ± 0.006 b	0.231 ± 0.011 a	0.109 ± 0.027 a	0.107 ± 0.029 b	0.058 ± 0.008 a	0.318 ± 0.009 ab
N_TL	0.126 ± 0.032 a	0.197 ± 0.037 a	0.297 ± 0.015 ab	0.217 ± 0.028 a	0.059 ± 0.018 bc	0.132 ± 0.031 ab	0.068 ± 0.009 a	0.324 ± 0.018 ab
NPK_TL	0.126 ± 0.020 a	0.207 ± 0.016 a	0.299 ± 0.004 ab	0.217 ± 0.009 a	0.075 ± 0.004 b	0.131 ± 0.020 ab	0.062 ± 0.006 a	0.327 ± 0.007 ab
M_TL	0.113 ± 0.017 a	0.195 ± 0.023 a	0.302 ± 0.016 ab	0.224 ± 0.027 a	0.088 ± 0.015 ab	0.118 ± 0.016 ab	0.060 ± 0.003 a	0.317 ± 0.010 b
MN_TL	0.116 ± 0.039 a	0.196 ± 0.050 a	0.319 ± 0.021 a	0.195 ± 0.019 a	0.044 ± 0.014 c	0.139 ± 0.047 ab	0.064 ± 0.017 a	0.339 ± 0.012 a
MNPK_TL	0.143 ± 0.013 a	0.224 ± 0.017 a	0.304 ± 0.010 ab	0.209 ± 0.022 a	0.060 ± 0.009 bc	0.166 ± 0.026 a	0.073 ± 0.007 a	0.337 ± 0.004 ab
Treatment	ns	ns	*	ns	*	ns	ns	ns
Depth	***	***	***	***	***	***	***	***
Treatments × Depth	ns	ns	ns	ns	**	ns	ns	ns

Data represent means ± SD (n = 3). Means in each column not sharing any lowercase letters at the same soil layer are significantly different (*p* < 0.05, Duncan’s test). ns—not significant; *—*p* < 0.05; **—*p* < 0.01; ***—*p* < 0.001 (LSD); CK—no fertilization; N—only nitrogen fertilizer; NPK—nitrogen, phosphorus, and potassium fertilizers; M—only organic fertilizer; MN—N + M; MNPK—NPK + M; FL—0–20 cm soil layer; SL—20–40 cm soil layer; TL—40–60 cm soil layer.

**Table 5:** Phosphorus cycle-related microbial functional gene abundances under different fertilization treatments and soil depths.

Treatments × Depth	Polyphosphate Consolidation	Polyphosphate Degradation	P Starvation Response Regulation	P Uptake and Transport System	Inorganic P Solubilization	Organic P Mineralization
CK_FL	0.028 ± 0.001 c	0.211 ± 0.008 ab	0.617 ± 0.016 abc	0.697 ± 0.024 ab	0.162 ± 0.006 abc	0.615 ± 0.008 ab
N_FL	0.030 ± 0.001 ab	0.214 ± 0.004 ab	0.627 ± 0.032 a	0.673 ± 0.006 b	0.169 ± 0.004 a	0.598 ± 0.007 b
NPK_FL	0.030 ± 0.000 abc	0.217 ± 0.005 a	0.603 ± 0.011 abc	0.701 ± 0.007 ab	0.167 ± 0.002 ab	0.611 ± 0.005 ab
M_FL	0.031 ± 0.002 a	0.217 ± 0.006 a	0.620 ± 0.011 ab	0.674 ± 0.023 b	0.160 ± 0.006 bc	0.625 ± 0.023 a
MN_FL	0.029 ± 0.001 abc	0.207 ± 0.002 b	0.590 ± 0.008 bc	0.725 ± 0.001 a	0.159 ± 0.002 bc	0.617 ± 0.007 ab
MNPK_FL	0.028 ± 0.001 bc	0.217 ± 0.001 ab	0.587 ± 0.009 c	0.699 ± 0.027 ab	0.156 ± 0.006 c	0.616 ± 0.007 ab
CK_SL	0.029 ± 0.003 a	0.211 ± 0.004 b	0.637 ± 0.011 a	0.653 ± 0.028 a	0.162 ± 0.019 a	0.601 ± 0.008 a
N_SL	0.027 ± 0.001 a	0.223 ± 0.009 a	0.619 ± 0.006 a	0.639 ± 0.010 a	0.176 ± 0.011 a	0.581 ± 0.009 a
NPK_SL	0.029 ± 0.005 a	0.215 ± 0.001 ab	0.621 ± 0.023 a	0.639 ± 0.027 a	0.184 ± 0.013 a	0.590 ± 0.002 a
M_SL	0.027 ± 0.001 a	0.219 ± 0.004 ab	0.626 ± 0.025 a	0.667 ± 0.026 a	0.165 ± 0.005 a	0.591 ± 0.023 a
MN_SL	0.029 ± 0.001 a	0.219 ± 0.001 ab	0.606 ± 0.027 a	0.673 ± 0.009 a	0.176 ± 0.013 a	0.590 ± 0.034 a
MNPK_SL	0.027 ± 0.002 a	0.223 ± 0.002 a	0.594 ± 0.031 a	0.655 ± 0.019 a	0.165 ± 0.013 a	0.596 ± 0.039 a
CK_TL	0.029 ± 0.004 a	0.214 ± 0.004 bc	0.640 ± 0.025 a	0.673 ± 0.009 a	0.155 ± 0.007 c	0.560 ± 0.011 a
N_TL	0.026 ± 0.002 ab	0.221 ± 0.007 ab	0.636 ± 0.005 a	0.673 ± 0.006 a	0.166 ± 0.010 bc	0.557 ± 0.024 a
NPK_TL	0.024 ± 0.003 ab	0.211 ± 0.001 c	0.620 ± 0.009 a	0.675 ± 0.011 a	0.167 ± 0.010 bc	0.550 ± 0.020 a
M_TL	0.025 ± 0.004 ab	0.217 ± 0.003 abc	0.601 ± 0.034 ab	0.704 ± 0.060 a	0.154 ± 0.006 c	0.554 ± 0.025 a
MN_TL	0.022 ± 0.005 ab	0.223 ± 0.005 a	0.566 ± 0.019 bc	0.690 ± 0.025 a	0.183 ± 0.011 a	0.553 ± 0.037 a
MNPK_TL	0.019 ± 0.003 b	0.222 ± 0.001 ab	0.559 ± 0.026 c	0.709 ± 0.040 a	0.172 ± 0.006 ab	0.531 ± 0.019 a
Treatment	ns	**	***	ns	**	ns
Depth	***	**	ns	***	*	***
Treatments × Depth	ns	*	ns	ns	ns	ns

Data represent means ± SD (n = 3). Means in each column not sharing any lowercase letters at the same soil layer are significantly different (*p* < 0.05, Duncan’s test). ns—not significant; *—*p* < 0.05; **—*p* < 0.01; ***—*p* < 0.001 (LSD); CK—no fertilization; N—only nitrogen fertilizer; NPK—nitrogen, phosphorus, and potassium fertilizers; M—only organic fertilizer; MN—N + M; MNPK—NPK + M; FL—0–20 cm soil layer; SL—20–40 cm soil layer; TL—40–60 cm soil layer.

## 4. Discussion

### 4.1. Interactive Effects of Fertilization and Soil Depth on Soil Chemical Properties and Rice Yield

The pronounced positive effects of organic fertilizer inputs on soil fertility and rice productivity observed in this study are consistent with the well-established role of organic amendments in improving nutrient availability and biological activity in surface soils. Under long-term no-tillage conditions in this study, the benefits of organic and combined fertilization were largely confined to the topsoil, highlighting a strong depth-dependent retention of nutrients and organic matter. Similar vertical constraints on nutrient redistribution have been reported in long-term fertilization experiments, where limited downward transport restricts fertilization effects primarily to surface layers [49,50]. The addition of organic fertilizers markedly elevates MBC in topsoil by supplying readily decomposable carbon substrates for soil microorganisms, such as simple sugars, organic acids, and amino acids, that stimulate microbial growth and metabolic activity [51,52], especially in organic and chemical fertilizer-combined treatments. Enhanced MBC drives accelerated nutrient cycling via upregulated extracellular enzyme activity, promoting faster nitrogen and phosphorus mineralization and improving plant nutrient availability [53]. However, the observed increases in soil electrical conductivity under manure-added treatments suggest that greater soluble salt accumulation might be attributed to the high soluble salt content in livestock manure [54,55]. These findings indicate that organic fertilization enhances surface soil fertility and crop productivity, but requires careful management to avoid potential salinity risks.

### 4.2. Soil Depth as the Primary Driver of Microbial Diversity and Community Assembly

Despite pronounced shifts in topsoil fertility, microbial α-diversity indices (Chao1, Shannon, Simpson, PD) showed no significant differences across long-term fertilization treatments but declined sharply with depth, corresponding with a previous report [56,57]. These results indicate that intrinsic heterogeneity in soil depths, specifically gradients in oxygen diffusion, moisture availability, and patterns of root exudation, plays a more decisive role in governing microbial species richness and evenness than do external nutrient inputs [21,26]. Rice typically develops its root network within the uppermost soil layers due to high water and nutrient availability [58], which provides a diverse array of root exudates, such as sugars, amino acids, flavonoids, aliphatic acids, proteins, and fatty acids [59]. These root metabolites have a more pronounced and direct influence on microbial α-diversity, β-diversity, and microbial community structure than soil physicochemical factors [60]. It is noteworthy that a more extensive root system does not necessarily translate into higher rhizosphere microbial diversity, suggesting that root–microbe interactions are intricately governed by the complex quality and composition of root exudates [61]. Microbial β-diversity analyses showed that surface soil microbial assemblages under organic-added treatments clustered separately from inorganic and unfertilized controls. This result indicates that soil microbial β-diversity is highly responsive to fertilization practices, with organic amendments markedly reshaping community structure by increasing total nutrient content and availability. However, the subsoil microbial communities showed substantial overlap among treatments and instead were grouped primarily by soil depth, with soil chemical factors explaining only a small fraction of the variation and not reaching statistical significance. These results showed weak soil microbial responses to fertilization and high convergence in deep layers. They further highlight soil depth as the dominant factor structuring microbial communities and the relative stability of deep-soil assemblages against surface management disturbances [24].

Although there was substantial nutrient enrichment in surface soils under organic and combined fertilization, dominant microbial taxa at the phylum and class levels remained largely unchanged, with only minor responses observed in a few groups. This phenomenon aligns with previous findings that chemical fertilizer, organic fertilizer, or their combination mediated the overall composition of dominant microbial phyla and classes remained largely unchanged, with only slight shifts in the relative abundances of certain copiotrophic or oligotrophic taxa [28,62]. However, with increasing soil depth, the relative abundances of most bacterial phyla and classes significantly declined, whereas the phyla Pseudomonadota, Methylomirabilota, and Nitrospirota and their corresponding classes Gammaproteobacteria, Methylomirabilia, and Nitrospiria became significantly enriched in deeper soils, which is in agreement with previous reports [63,64,65]. These results indicated that the functional adaptations of deep-soil microbial communities are a key driver of their vertical distribution patterns. Their enrichment in deeper soils reflects their roles as facultative anaerobes and chemolithoautotrophs, respectively, capable of using nitrate/nitrite, iron and manganese oxides, or methane as terminal electron acceptors to sustain energy metabolism under subsoil conditions [66,67]. Microbial responses to fertilization were most pronounced in surface soils, where a greater number of discriminative taxa were identified compared with deeper soil layers. The surface soil is enriched in soluble organic matter, root exudates, and oxygen, creating a diverse ecological niche that supports the coexistence and alternation of copiotrophic and oligotrophic microbial groups [68,69], thus leading to a greater number of significant biomarkers. In contrast, with increasing soil depth, weakened root influence, depleted organic carbon, and reduced oxygen impose stronger environmental filtering on the subsoil microbiome, allowing only a few specialist or facultative anaerobic taxa adapted to various restrictive conditions such as low-nutrient, low-oxygen, or other abiotic stresses to persist [57,70,71], thereby decreasing the number of biomarkers specific to different fertilization treatments.

### 4.3. The Characteristics of Microbial Co-Occurrence Networks and Nutrient Cycling Functional Gene Distributions Driven by Soil Depth and Fertilization

In the topsoil, the microbial co-occurrence network exhibited the highest complexity, indicating the presence of extensive synergistic and potentially mutualistic relationships within the microbial community. This structurally complex and cooperative network coincided with the highest abundance of functional genes involved in the decomposition of recalcitrant organic matter such as lignin, cellulose, and chitin. In addition, functional genes related to C, N, and P cycling showed active responses to different fertilization treatments in the topsoil, highlighting soil microbial functional plasticity in nutrient cycling and soil multifunctionality under agricultural interventions [72]. In contrast, the microbial network in the middle and deep layers was less connected and clustered, with reduced node and edge numbers. However, a higher proportion of positive correlations was found, especially in the 40–60 cm soil layer, suggesting a shift toward more tightly cooperative but less diverse microbial assemblages. This is in agreement with the findings of Liu et al. [57], who also observed increased positive links in bacterial networks in deeper soil layers, likely due to resource scarcity and stronger physical constraints that promote cooperative relationships to optimize resource utilization and enhance survival. This network simplification was accompanied by a decline in C cycling functional gene abundances involved in organic matter decomposition. In contrast, functional genes abundances related to deeper-soil C and N cycling processes, such as C fixation, methanogenesis, denitrification, and anaerobic ammonium oxidation became more abundant. These observations indicate that, although the microbial community structure becomes simplified with soil depth it gradually shifts toward anaerobic metabolic functions and a conservative metabolic strategy. This emphasizes microbial survival adaption under low-energy and low-nutrient conditions, highlighting that deeper-soil microbial communities still hold significant ecological importance despite their low abundance [5,73,74].

Compared with soil depth, fertilization played a relatively minor role in affecting the abundances of microbial functional genes involved in carbon, nitrogen, and phosphorus cycling. For instance, although N fixation functional genes were significantly increased under organic fertilization alone, such effects were not evident under combined organic–inorganic fertilization treatments, suggesting that the input of inorganic nitrogen may suppress energy-intensive N fixation processes [75,76,77]. Moreover, in the absence of fertilization or under mineral fertilization treatments, the microbial activity remained low due to the lack of sufficient C sources and other essential nutrients, thus resulting in deficiencies in the reducing power and energy necessary for N fixation [78,79]. With respect to phosphorus cycling, the abundance of functional genes related to P starvation response regulation and inorganic P solubilization was lower in treatments with organic fertilizer addition, especially under the combination of organic and chemical fertilizers [80,81]. This may be attributed to a more sustained and slow P release of organic fertilizer, reducing microbial P acquisition stress and thus the need for upregulating P starvation response and inorganic P solubilization functional genes [82]. This limited fertilization response is likely due to the high microbial network complexity and functional redundancy observed in the topsoil, where the co-occurrence network exhibited the highest node density and connectivity [83,84]. These results imply strong cooperative interactions and a high prevalence of functionally similar microbial taxa. Such community organization could maintain the relative stability of C, N, and P cycling processes through functional complementarity and substitution, thereby exhibiting strong resistance to fertilization disturbance and high adaptability to environmental changes [85,86,87].

## 5. Conclusions

Results from this 40-year field experiment demonstrate that long-term fertilization significantly altered soil chemical properties, with pronounced increases in SOM, TN, TP, AP, and MBC in the topsoil (0–20 cm), particularly under organic and combined organic–inorganic fertilization. These improvements were accompanied by increased rice yields. In contrast, fertilization effects on soil fertility indicators were substantially weaker in subsoil layers (20–40 cm and 40–60 cm), indicating limited downward transfer of nutrients. Soil microbial alpha diversity showed minimal response to fertilization but declined markedly with increasing soil depth, reflecting strong vertical stratification. Microbial community composition and structure were primarily driven by soil depth, whereas fertilization effects were mainly confined to surface soils. Microbial co-occurrence networks exhibited the highest complexity and connectivity in topsoil and became progressively simpler with depth. Functional gene profiles also displayed clear depth-dependent patterns, with surface soils enriched in decomposition-related genes and deeper soils dominated by anaerobic pathways. Overall, these findings identify soil depth as a dominant factor shaping microbial community structure and functional potential, while fertilization primarily regulates soil fertility and microbial activity in surface soils. Improving surface soil fertility while maintaining microbial stability in deeper layers is essential for sustaining crop productivity.

## Data Availability

The sequencing raw data were deposited in the NCBI Short Read Archive (SRA) database under accession number PRJNA1304740.

## Supplementary Materials

The following supporting information can be downloaded at https://www.mdpi.com/article/10.3390/microorganisms14010250/s1, Table S1: The variance in microbial communities that each soil environmental variable can independently explain under different soil layers; Table S2: Relative abundances of dominant bacterial phyla across fertilization treatments and soil depths; Table S3: Relative abundances of dominant bacterial classes across fertilization treatments and soil depths; Table S4: Soil microbial KEGG pathways that related to carbon, nitrogen, and phosphorus cycles. Figure S1: LEfSe cladograms showing significantly abundant microbial community taxa among different fertilization treatments in the first (0–20 cm, a), second (20–40 cm, b), and third (40–60 cm, c) soil layers. Figure S2: Linear discriminant analysis coupled with effect size measurements identifies the different abundant microbial community taxa in the first (0–20 cm, a), second (20–40 cm, b), and third (40–60 cm, c) soil layers among different fertilization treatments.
