# Supplementary material for: Long-Term Organic Fertilization Enhances Soil Fertility and Reshapes Microbial Community Structure with Decreasing Effects Across Soil Depth
Source: Microorganisms. 2026 Jan 21;14(1):250. doi: 10.3390/microorganisms14010250 (PMC12843692; doi:10.3390/microorganisms14010250)
Supplement: Supplementary file 1 [file microorganisms-14-00250-s001.zip › microorganisms-4069703-supplementary.pdf]

Long-Term Organic Fertilization Enhances Soil Fertility and Reshapes Microbial Community Structure with Decreasing Effects Across Soil Depth

Suyao Li [1,†], Yulin Li [1,†], Xu Yan [1], Zhengyang Gu [1], Dong Xue [2], Kaihua Wang [2], Yuting Yang [1], Min Lv [1], Yujie Han [1], Jinbiao Li [1], Yanyan Lv [1,*] and Anyong Hu [1,*]

[1] School of Geographical Science, Nantong University, Nantong 226019, China; 19517128836@163.com (S.L.); yulinli922@sina.com (Y.L.); yanxu20050129@sina.com (X.Y.); gzy13815927234@163.com (Z.G.); ytyang@ntu.edu.cn (Y.Y.); lm2018@ntu.edu.cn (M.L.); hanyj@ntu.edu.cn (Y.H.); jbli@ntu.edu.cn (J.L.)

[2] Jiangsu Yanjiang Institute of Agricultural Sciences, Nantong 226541, China; xuedongjsrg@jaas.ac.cn (D.X.); 19942003@jaas.ac.cn (K.W.)

* Correspondence: lvyy18@ntu.edu.cn (Y.L.); ayhu2018@ntu.edu.cn (A.H.)

† S.L. and Y.L. contributed equally to this work.

Table S1. The variance in microbial communities that each soil environmental variable can independently explain under different soil layers.

First layer (0–20 cm)		Second layer (20–40 cm)		Third layer (40–60 cm)		All layer (0–60 cm)	
Variables	Explained variation (%)	Variables	Explained variation (%)	Variables	Explained variation (%)	Variables	Explained variation (%)
TN	3.82*	EC	2.98	WSOC	2.71	TN	10.85*
WSOC	3.76*	TN	2.68	AK	1.90	SOM	10.76*
AP	3.70*	SOM	2.60	TN	1.87	WSOC	10.19*
TP	3.61*	AP	1.84	SOM	1.67	AK	6.42*
SOM	3.59*	NH_4^+-N	1.82	TP	1.58	TP	5.80*
EC	2.81*	AK	1.69	EC	1.58	AP	5.76*
AK	2.71*	TP	1.67	NO_3^--N	1.18	NH_4^+-N	3.06*
pH	1.36	WSOC	1.63	pH	1.06	pH	2.14*
NO_3^--N	1.22	NO_3^--N	1.29	NH_4^+-N	1.00	EC	1.21
NH_4^+-N	1.04	pH	1.08	AP	0.92	NO_3^--N	0.68

Note: * P < 0.05. EC, electrical conductivity; SOM, soil organic matter; WSOC, water-soluble organic carbon; TN, total nitrogen; TP, total phosphorus; AP, available phosphorus; AK, available potassium.

Table S2. Relative abundances of dominant bacterial phyla across fertilization treatments and soil depths.

Treatments × Depth	Acidobacteriota	Pseudomonadota	Chloroflexota	Methylomirabilota	Nitrospirota	Bacillota	Actinomycetota	Planctomycetota	Myxococcota	Thermodesulfo bacteriota	Gemmatimonadota	MBNT15	Others
CK_FL	18.79±2.05a	14.43±0.50a	15.69±2.60a	3.44±0.70a	4.99±0.39a	6.42±1.01a	3.58±0.83ab	3.63±0.51a	4.08±0.64ab	2.34±0.48b	3.22±0.92a	1.78±0.64ab	17.62±1.09a
N_FL	18.31±1.43a	12.93±3.25a	14.97±1.26a	1.98±0.33ab	5.76±0.59a	8.09±1.75a	4.86±0.48ab	3.34±0.50ab	3.89±0.20ab	2.33±0.53b	2.88±0.36ab	1.40±0.35ab	19.26±3.18a
NPK_FL	16.86±1.03a	13.98±2.59a	15.61±0.91a	2.16±0.94ab	6.04±0.55a	6.26±1.38a	4.43±0.94ab	2.60±0.69ab	4.42±1.03a	2.64±0.36ab	2.05±0.44b	0.96±0.26b	21.99±3.02a
M_FL	17.76±0.82a	15.13±2.15a	14.05±1.21a	2.43±1.50ab	6.15±0.79a	6.10±2.19a	2.96±1.05b	3.06±0.90ab	3.78±0.69ab	3.60±0.80a	2.90±0.45ab	1.77±0.66ab	20.30±3.25a
MN_FL	17.82±2.71a	16.24±1.88a	12.75±1.21a	1.87±0.61ab	5.09±1.29a	8.23±2.92a	6.36±2.45a	2.19±0.71b	3.27±0.09b	2.41±0.41ab	2.51±0.48ab	1.80±0.59ab	19.46±2.50a
MNPK_FL	17.67±1.61a	15.62±2.39a	13.45±1.97a	1.40±0.33b	5.50±0.73a	7.00±1.52a	4.86±2.34ab	2.68±0.88ab	3.35±0.11ab	2.45±0.95ab	2.53±0.46ab	2.27±0.82a	21.23±4.30a
CK_SL	17.80±0.98a	16.33±0.41a	13.76±1.34bc	9.04±0.14a	6.55±0.88a	4.93±0.81a	1.75±0.59a	3.38±0.28a	2.64±0.39a	2.24±0.47a	1.68±0.72b	1.74±0.17a	18.16±1.07ab
N_SL	17.88±2.31a	17.17±4.48a	16.35±0.29ab	7.24±3.30a	5.19±1.38a	3.62±2.20a	2.62±0.61a	3.35±0.30a	2.10±0.81a	1.59±0.71a	2.87±0.59ab	2.55±1.61a	17.46±2.21ab
NPK_SL	17.48±0.57a	17.00±1.74a	18.12±1.89a	8.89±3.82a	6.44±2.05a	2.60±0.72a	1.91±0.12a	3.06±0.54a	1.97±0.74a	2.05±0.78a	2.83±0.32ab	2.42±0.70a	15.22±1.93b
M_SL	17.89±0.77a	16.02±2.61a	13.69±0.87bc	5.36±2.38a	6.58±1.03a	5.19±1.56a	2.52±1.05a	2.87±0.71a	3.00±0.51a	2.15±0.73a	3.38±1.47a	2.30±0.23a	19.05±2.10ab
MN_SL	17.28±1.48a	17.54±2.35a	12.83±1.67c	6.77±4.87a	5.55±0.68a	4.51±1.73a	2.34±0.93a	3.07±0.15a	2.45±0.70a	3.66±2.84a	2.58±0.70ab	2.17±1.03a	19.24±5.97ab
MNPK_SL	17.34±0.73a	16.23±3.29a	14.50±2.88bc	5.96±5.38a	5.00±0.86a	3.69±1.39a	2.12±0.43a	3.00±0.94a	2.51±0.72a	2.76±1.19a	2.57±0.57ab	2.48±0.93a	21.85±1.85a
CK_TL	14.93±0.68a	16.17±1.20a	12.40±1.94a	16.08±1.00a	7.33±0.32a	2.74±0.62a	1.57±0.29a	2.45±0.32ab	2.16±0.31a	4.40±1.46a	1.67±0.20b	0.69±0.26b	17.41±3.14a
N_TL	15.63±1.30a	16.73±0.62a	12.19±1.88a	10.50±5.57b	8.26±0.99a	4.89±2.93a	2.37±0.29a	3.06±0.61a	2.36±1.05a	2.54±0.96a	2.09±0.20ab	1.29±0.46ab	18.09±2.43a
NPK_TL	14.93±1.18a	16.81±1.69a	13.97±0.47a	16.51±1.31a	8.00±0.96a	2.96±1.31a	2.06±0.31a	1.87±0.26b	1.94±0.20a	2.49±0.07a	1.77±0.17b	0.99±0.33b	15.70±0.49a
M_TL	14.91±1.01a	17.72±1.40a	12.61±1.86a	15.19±2.31ab	7.76±1.09a	2.24±0.92a	1.94±0.73a	2.27±0.25b	1.92±0.62a	4.02±0.30a	2.07±0.11ab	0.96±0.24b	16.38±0.58a
MN_TL	15.37±1.12a	18.74±3.63a	12.01±2.05a	11.73±2.71ab	6.91±1.06a	3.64±1.01a	2.37±0.51a	2.34±0.24b	2.31±0.58a	2.59±1.75a	2.69±0.72a	1.89±0.80a	17.41±1.33a
MNPK_TL	15.67±0.24a	17.96±0.72a	12.31±0.93a	14.77±1.39ab	8.07±0.96a	2.25±0.87a	2.11±0.51a	2.24±0.45b	1.86±0.13a	2.78±0.29a	2.02±0.48ab	0.84±0.50b	17.11±1.05a
Treatments	ns	ns	**	ns	ns	ns	ns	*	ns	ns	ns	ns	ns
Soil depth	***	**	***	***	***	***	***	**	***	ns	**	***	**
Treatments × Depth	ns	ns	ns	ns	ns	ns	ns	ns	ns	ns	ns	ns	ns

Data represent means ± SD (n = 3). Means in each column not sharing any lowercase letters at the same soil layer are significantly different ($P < 0.05$, Duncan's test). ns: not significant; *, $P < 0.05$; **, $P < 0.01$; ***, $P < 0.001$ (LSD). CK, no fertilization; N, only nitrogen fertilizer; NPK, nitrogen, phosphorus and potassium fertilizers; M, only organic fertilizer; MN, N+M; MNPK, NPK+M. FL, 0–20 cm soil layer; SL, 20–40 cm soil layer; TL, 40–60 cm soil layer.

Table S3. Relative abundances of dominant bacterial classes across fertilization treatments and soil depths.

Treatments × Depth	Anaerolineae	Gammaproteobacteria	Vicinamibacteria	Methylomirabilia	Alphaproteobacteria	Nitrospiria	Bacilli	bacteriap25	Acidobacteriae	Planctomycetes	Gemmatimonadia	MBNT15	Others
CK_FL	13.59±2.61a	8.56±0.51a	12.15±1.67a	3.44±0.70a	5.88±0.65ab	3.51±0.19b	5.53±0.62a	3.57±0.38a	1.41±0.39a	2.33±0.14a	2.24±0.62a	1.78±0.64ab	36.02±0.62b
N_FL	12.95±1.01ab	7.09±2.25a	10.72±1.20a	1.98±0.33ab	5.84±1.07ab	4.94±0.14a	7.39±1.93a	3.43±0.05a	1.01±0.22ab	2.44±0.07a	1.76±0.47ab	1.40±0.35ab	39.05±2.42ab
NPK_FL	13.47±0.69a	7.93±1.83a	10.48±0.33a	2.16±0.94ab	6.04±0.80ab	4.48±0.54a	5.63±1.25a	3.88±1.20a	0.59±0.63ab	1.81±0.59a	1.16±0.66b	0.96±0.26b	41.41±3.18a
M_FL	12.30±1.03ab	10.12±2.25a	11.00±0.58a	2.43±1.50ab	5.01±0.33b	4.39±0.26a	5.88±2.17a	3.18±0.83a	0.80±0.42ab	2.18±0.65a	2.09±0.46ab	1.77±0.66ab	38.87±2.69ab
MN_FL	10.36±1.20b	9.67±1.83a	12.03±2.68a	1.87±0.61ab	6.58±0.05a	4.25±0.76ab	7.93±2.80a	2.99±0.21a	0.39±0.34b	1.49±0.46a	1.53±0.18ab	1.80±0.59ab	39.12±0.46ab
MNPK_FL	11.75±1.39ab	9.80±2.42a	11.47±0.74a	1.40±0.33b	5.82±0.08ab	4.36±0.49a	6.63±1.64a	3.07±0.20a	0.56±0.46b	1.67±0.69a	1.85±0.55ab	2.27±0.82a	39.36±3.84ab
CK_SL	12.36±0.91bc	11.50±0.94a	10.71±0.34a	9.04±0.14a	4.84±0.53a	4.88±1.15a	4.23±0.82a	2.23±0.22ab	2.32±0.37ab	2.46±0.18a	1.22±0.75a	1.74±0.17a	32.48±0.28a
N_SL	14.85±0.44ab	11.88±2.94a	10.28±0.84a	7.24±3.30a	5.30±1.58a	3.61±1.51a	3.00±2.04a	1.70±0.73ab	3.16±1.61ab	2.48±0.07a	2.19±0.72a	2.55±1.61a	31.75±3.63a
NPK_SL	16.37±2.24a	12.70±1.56a	9.43±0.98a	8.89±3.82a	4.31±0.19a	3.86±1.70a	2.19±0.84a	1.41±0.32b	3.88±1.00a	2.33±0.41a	2.52±0.35a	2.42±0.70a	29.69±2.24a
M_SL	11.93±0.15bc	10.97±3.00a	10.87±0.48a	5.36±2.38a	5.04±0.50a	4.52±0.81a	4.60±1.44a	2.54±0.46a	1.69±0.50b	1.92±0.47a	2.62±1.35a	2.30±0.23a	35.63±3.67a
MN_SL	11.40±1.34c	12.85±2.83a	10.34±1.13a	6.77±4.87a	4.69±0.50a	3.82±1.02a	3.87±1.42a	2.22±0.64ab	1.81±0.38ab	2.13±0.16a	1.82±0.45a	2.17±1.03a	36.11±7.67a
MNPK_SL	13.32±2.53bc	11.28±2.28a	10.66±0.89a	5.96±5.38a	4.94±1.02a	3.20±0.42a	3.26±1.27a	2.13±0.63ab	1.82±1.64ab	1.90±0.71a	2.00±0.60a	2.48±0.93a	37.02±4.32a
CK_TL	10.43±1.94a	13.05±1.11a	7.23±0.33a	16.08±1.00a	3.11±0.11b	5.10±0.34a	2.30±0.43a	1.94±0.24a	3.97±0.61a	1.71±0.05b	1.52±0.20a	0.69±0.26b	32.85±2.73a
N_TL	10.36±1.90a	12.55±0.94a	8.23±0.91a	10.50±5.57b	4.18±1.30ab	6.81±0.93a	4.37±3.07a	2.01±1.04a	3.11±1.69a	2.22±0.35a	1.55±0.10a	1.29±0.46ab	32.81±3.03a
NPK_TL	11.94±0.36a	13.20±1.78a	7.57±0.78a	16.51±1.31a	3.61±0.31ab	5.86±0.94a	2.53±1.05a	1.76±0.39a	4.00±0.54a	1.56±0.25b	1.60±0.17a	0.99±0.33b	28.88±1.53a
M_TL	10.57±1.87a	13.80±2.11a	7.07±0.93a	15.19±2.31ab	3.92±0.80ab	6.17±0.72a	1.91±0.75a	1.78±0.64a	3.81±0.70a	1.72±0.25b	1.85±0.21a	0.96±0.24b	31.24±1.15a
MN_TL	10.37±1.84a	13.72±3.54a	7.79±0.40a	11.73±2.71ab	5.02±0.98a	5.20±1.61a	3.17±1.04a	2.25±0.54a	3.41±0.33a	1.77±0.13b	2.21±0.83a	1.89±0.80a	31.48±3.66a
MNPK_TL	10.25±0.94a	13.63±0.90a	7.54±0.07a	14.77±1.39ab	4.33±0.56ab	6.37±0.74a	1.76±0.81a	1.73±0.20a	3.89±0.39a	1.48±0.19b	1.79±0.46a	0.84±0.50b	31.63±1.68a
Treatment	**	ns	ns	ns	ns	ns	ns	ns	ns	**	ns	ns	ns
Depth	***	***	***	***	***	***	***	***	***	**	ns	***	***
Treatments × Depth	ns	ns	ns	ns	ns	ns	ns	ns	ns	ns	ns	ns	ns

Data represent means ± SD (n = 3). Means in each column not sharing any lowercase letters at the same soil layer are significantly different ($P < 0.05$, Duncan's test). ns: not significant; *, $P < 0.05$; **, $P < 0.01$; ***, $P < 0.001$ (LSD). CK, no fertilization; N, only nitrogen fertilizer; NPK, nitrogen, phosphorus and potassium fertilizers; M, only organic fertilizer; MN, N+M; MNPK, NPK+M. FL, 0–20 cm soil layer; SL, 20–40 cm soil layer; TL, 40–60 cm soil layer.

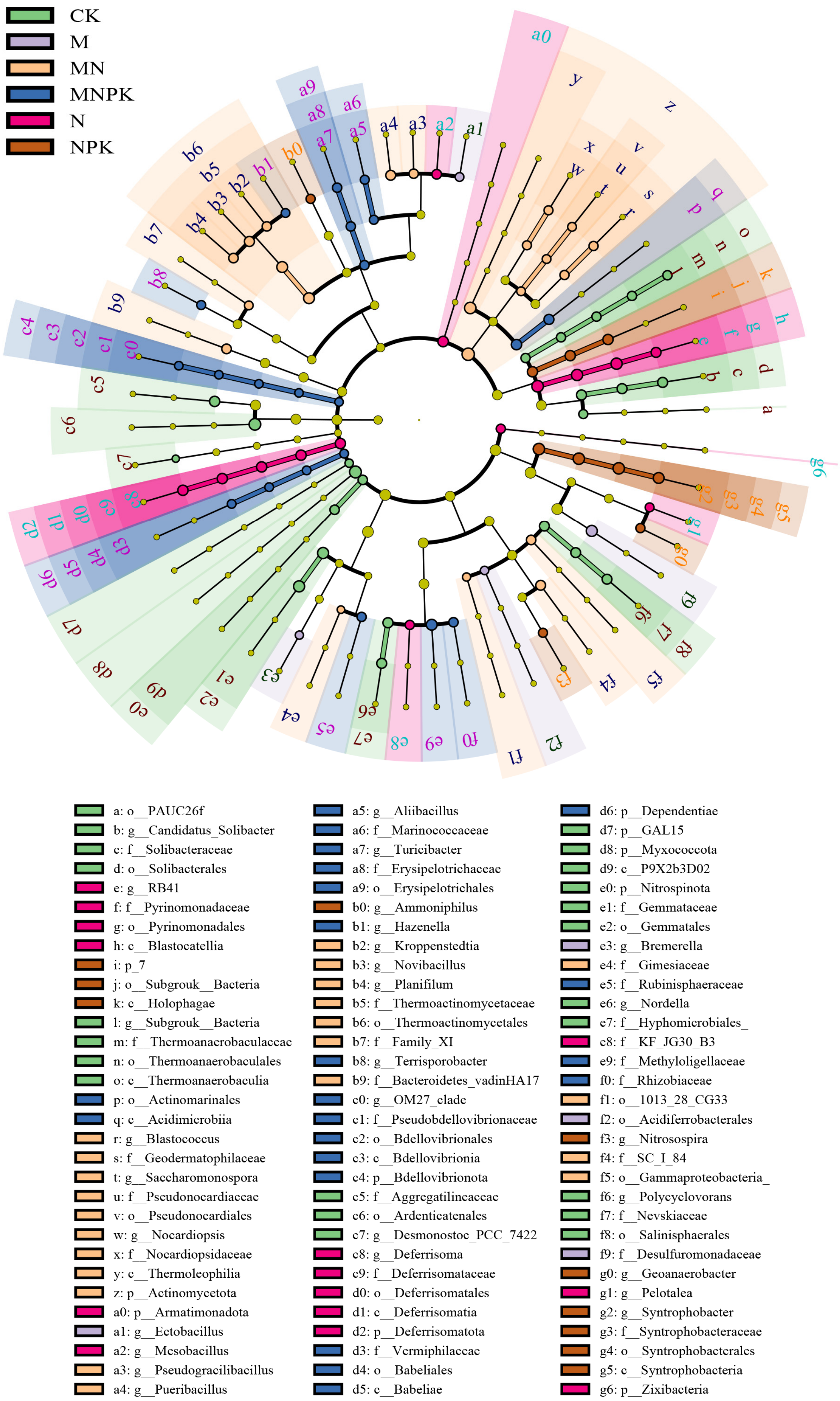

CK
M
MN
MNPK
N
NPK
a: o__PAUC26f
b: g__Candidatus_Solibacter
c: f__Solibacteraceae
d: o__Solibacterales
e: g__RB41
f: f__Pyrinomonadaceae
g: o__Pyrinomonadales
h: c__Blastocatellia
i: p_7
j: o__Subgrouk__Bacteria
k: c__Holophagae
l: g__Subgrouk__Bacteria
m: f__Thermoanaerobaculaceae
n: o__Thermoanaerobaculales
o: c__Thermoanaerobaculia
p: o__Actinomarinales
q: c__Acidimicrobiia
r: g__Blastococcus
s: f__Geodermatophilaceae
t: g__Saccharomonospora
u: f__Pseudonocardiaceae
v: o__Pseudonocardiales
w: g__Nocardiopsis
x: f__Nocardiopsidaceae
y: c__Thermoleophilia
z: p__Actinomycetota
a0: p__Armatimonadota
a1: g__Ectobacillus
a2: g__Mesobacillus
a3: g__Pseudogracilibacillus
a4: g__Pueribacillus
a5: g__Aliibacillus
a6: f__Marinococcaceae
a7: g__Turicibacter
a8: f__Erysipelotrichaceae
a9: o__Erysipelotrichales
b0: g__Ammoniphilus
b1: g__Hazenella
b2: g__Kroppenstedtia
b3: g__Novibacillus
b4: g__Planifilum
b5: f__Thermoactinomycetaceae
b6: o__Thermoactinomycetales
b7: f__Family_XI
b8: g__Terrisporobacter
b9: f__Bacteroidetes_vadinHA17
c0: g__OM27_clade
c1: f__Pseudobdellovibrionaceae
c2: o__Bdellovibrionales
c3: c__Bdellovibrionia
c4: p__Bdellovibrionota
c5: f__Aggregatilineaceae
c6: o__Ardenticatenales
c7: g__Desmonostoc_PCC_7422
c8: g__Deferrisoma
c9: f__Deferrisomataceae
d0: o__Deferrisomatales
d1: c__Deferrisomatia
d2: p__Deferrisomatota
d3: f__Vermiphilaceae
d4: o__Babeliales
d5: c__Babeliae
d6: p__Dependentiae
d7: p__GAL15
d8: p__Myxococcota
d9: c__P9X2b3D02
e0: p__Nitrospinota
e1: f__Gemmataceae
e2: o__Gemmatales
e3: g__Bremerella
e4: f__Gimesiaceae
e5: f__Rubinisphaeraceae
e6: g__Nordella
e7: f__Hyphomicrobiales_
e8: f__KF_JG30_B3
e9: f__Methyloligellaceae
f0: f__Rhizobiaceae
f1: o__1013_28_CG33
f2: o__Acidiferrobacterales
f3: g__Nitrosospira
f4: f__SC_I_84
f5: o__Gammaproteobacteria_
f6: g__Polycyclovorans
f7: f__Nevskiaceae
f8: o__Salinisphaerales
f9: f__Desulfuromonadaceae
g0: g__Geoanaerobacter
g1: g__Pelotalea
g2: g__Syntrophobacter
g3: f__Syntrophobacteraceae
g4: o__Syntrophobacterales
g5: c__Syntrophobacteria
g6: p__Zixibacteria

(a)

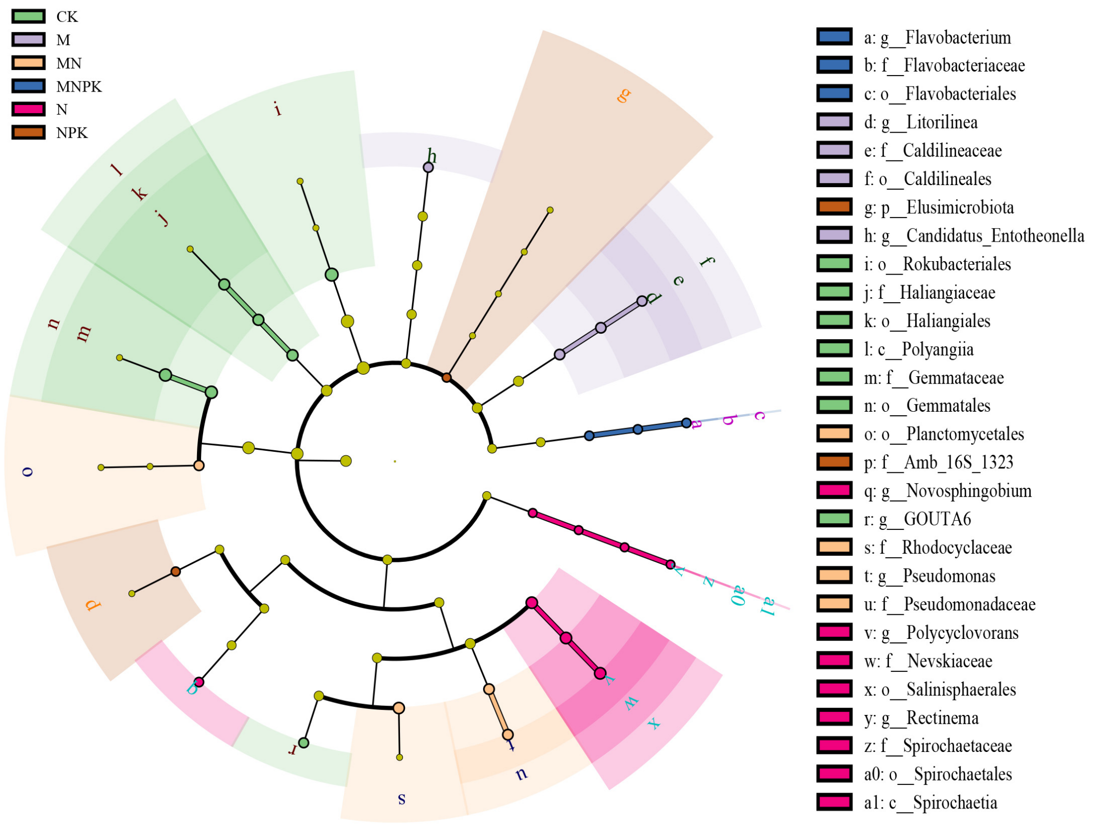

(**b**)

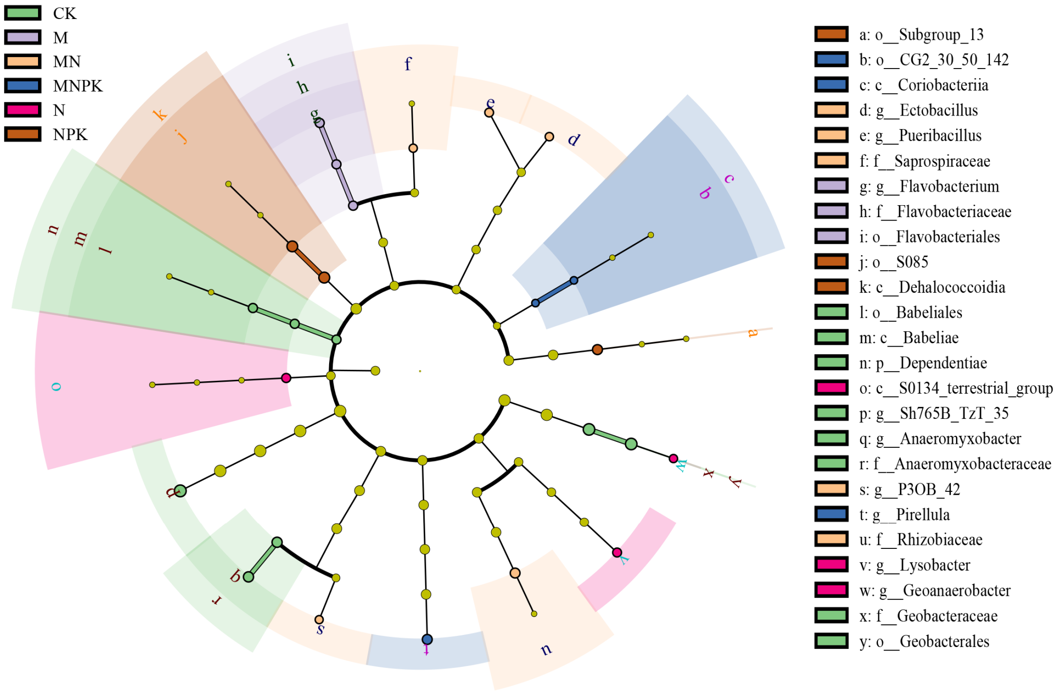

(**c**)

Figure S1. LEfSe cladograms showing significantly abundant microbial community taxa among different fertilization treatments in the first (0–20 cm, a), second (20–40 cm, b), and third (40–60 cm, c) soil layers. Taxa of different sites are represented by colored dots. Taxonomic cladograms ob-tained from only taxa meeting an LDA significance threshold of 2 are shown. Circles represent phylogenetic levels from kingdom to genus (outermost). Abbreviations: CK, no fertilization; N, only nitrogen fertilizer; NPK, nitrogen, phosphorus and potassium fertilizers; M, only organic fer-tilizer; MN, N + M; MNPK, NPK + M.

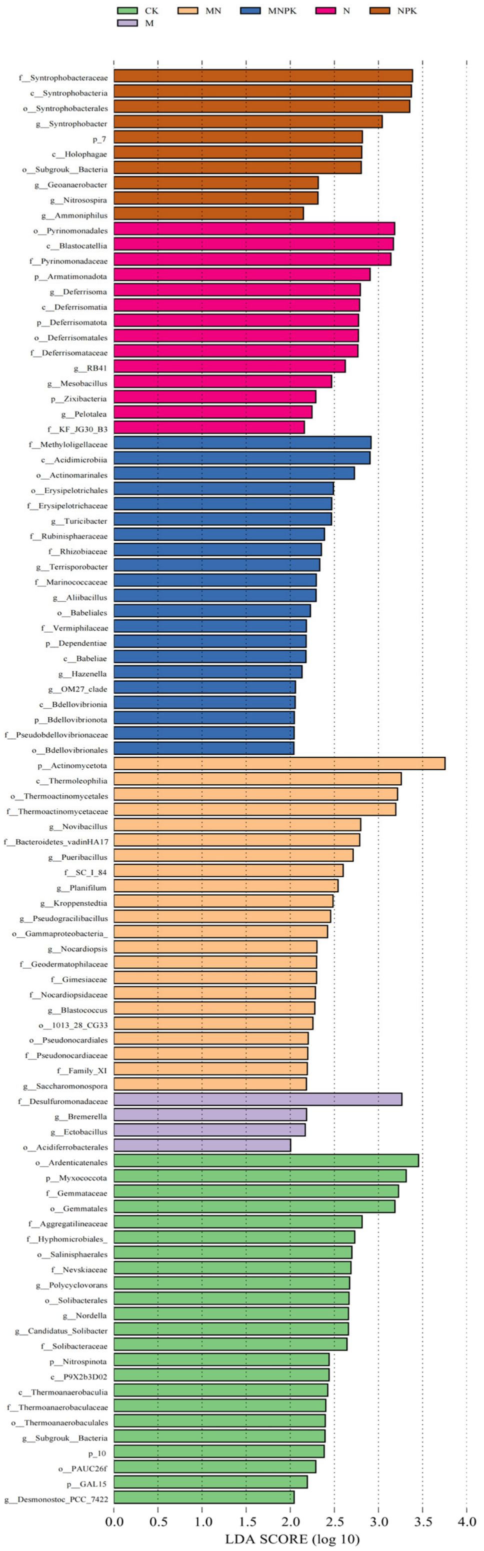

(**a**)

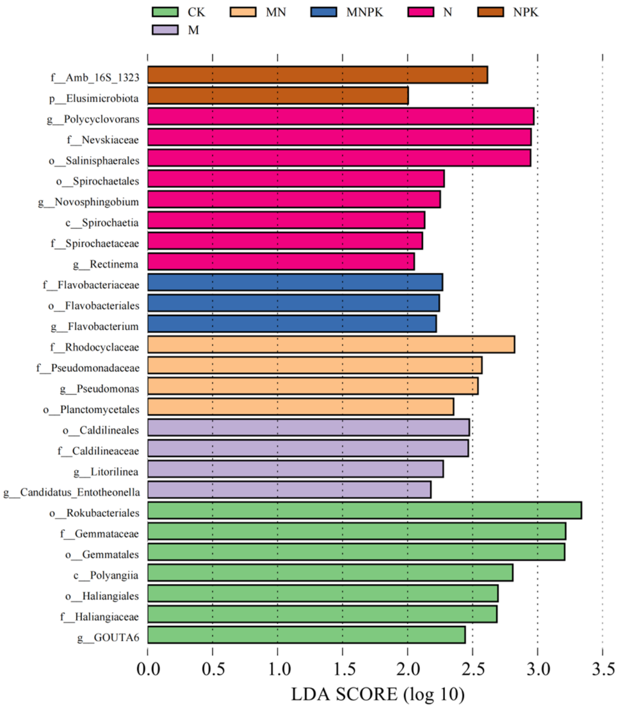

(**b**)

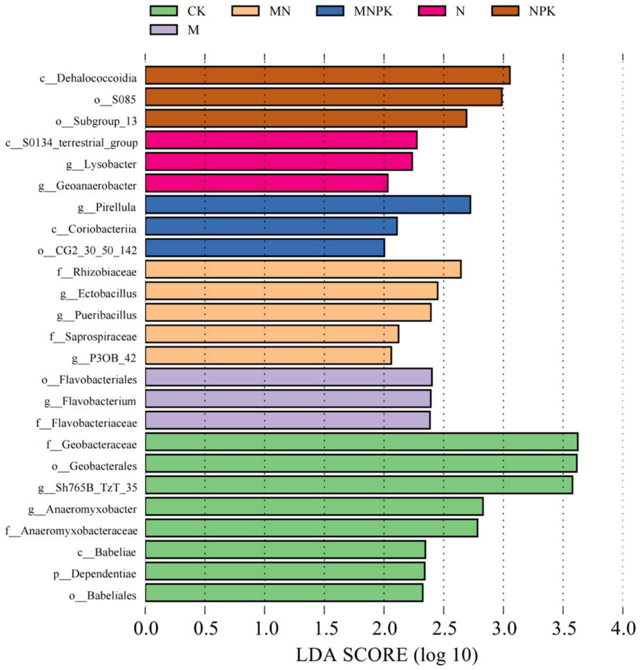

(**c**)

Figure S2. Linear discriminant analysis coupled with effect size measurements identifies the differ-ent abundant microbial community taxa in the first (0–20 cm, a), second (20–40 cm, b), and third (40–60 cm, c) soil layers among different fertilization treatments. Lineages with LDA values higher than 2.0 are displayed. Abbreviations: CK, no fertilization; N, only nitrogen fertilizer; NPK, nitro-gen, phosphorus and potassium fertilizers; M, only organic fertilizer; MN, N + M; MNPK, NPK + M.

Table S4. Soil microbial KEGG pathways that related to carbon, nitrogen, and phosphorus cycles.

Nutrient cycling	Function	KEGG pathway	Description
Carbon	Carbon fixation	K00024	malate dehydrogenase [EC:1.1.1.37]
		K00029	malate dehydrogenase (oxaloacetate-decarboxylating)(NADP+)
		K00031	isocitrate dehydrogenase [EC:1.1.1.42]
		K00134	glyceraldehyde 3-phosphate dehydrogenase
		K00150	glyceraldehyde-3-phosphate dehydrogenase (NAD(P)) 1.2.1.59
		K00169	pyruvate ferredoxin oxidoreductase alpha subunit [EC:1.2.7.1]
		K00170	pyruvate ferredoxin oxidoreductase beta subunit [EC:1.2.7.1]
		K00171	pyruvate ferredoxin oxidoreductase delta subunit [EC:1.2.7.1]
		K00172	pyruvate ferredoxin oxidoreductase gamma subunit [EC:1.2.7.1]
		K00174	2-oxoglutarate/2-oxoacid ferredoxin oxidoreductase subunit alpha [EC:1.2.7.3 1.2.7.11]
		K00175	2-oxoglutarate/2-oxoacid ferredoxin oxidoreductase subunit beta [EC:1.2.7.3 1.2.7.11]
		K00176	2-oxoglutarate ferredoxin oxidoreductase subunit delta [EC:1.2.7.3]
		K00177	2-oxoglutarate ferredoxin oxidoreductase subunit gamma [EC:1.2.7.3]
		K00194	acetyl-CoA decarbonylase/synthase complex subunit delta [EC:2.1.1.245]
		K00196	anaerobic carbon-monoxide dehydrogenase iron sulfur subunit
		K00197	acetyl-CoA decarbonylase/synthase complex subunit gamma [EC:2.1.1.245]
		K00198	anaerobic carbon-monoxide dehydrogenase catalytic subunit [EC:1.2.7.4]
		K00239	succinate dehydrogenase / fumarate reductase, flavoprotein subunit [EC:1.3.5.1]
		K00240	succinate dehydrogenase / fumarate reductase, iron-sulfur subunit [EC:1.3.5.1]
		K00241	succinate dehydrogenase / fumarate reductase, cytochrome b subunit
		K00242	succinate dehydrogenase / fumarate reductase, membrane anchor subunit
		K00244	fumarate reductase flavoprotein subunit [EC:1.3.5.4]
		K00297	methylenetetrahydrofolate reductase (NADPH) [EC:1.5.1.54]
		K00615	transketolase [EC:2.2.1.1]
		K00625	phosphate acetyltransferase [EC:2.3.1.8]
		K00626	acetyl-CoA C-acetyltransferase 2.3.1.9
		K00855	phosphoribulokinase [EC:2.7.1.19]
		K00925	acetate kinase [EC:2.7.2.1]
		K00927	phosphoglycerate kinase [EC:2.7.2.3]
		K01006	pyruvate, orthophosphate dikinase [EC:2.7.9.1]
		K01007	pyruvate, water dikinase [EC:2.7.9.2]
		K01086	fructose-1,6-bisphosphatase I / sedoheptulose-1,7-bisphosphatase [EC:3.1.3.11 3.1.3.37]
		K01491	methylenetetrahydrofolate dehydrogenase (NADP+) / methenyltetrahydrofolate cyclohydrolase [EC:1.5.1.5 3.5.4.9]
		K01499	methenyltetrahydromethanopterin cyclohydrolase [EC:3.5.4.27]
		K01595	phosphoenolpyruvate carboxylase [EC:4.1.1.31]
		K01601	ribulose-bisphosphate carboxylase large chain [EC:4.1.1.39]
		K01602	ribulose-bisphosphate carboxylase small chain [EC:4.1.1.39]
		K01610	phosphoenolpyruvate carboxykinase (ATP) [EC:4.1.1.49]

K01623	fructose-bisphosphate aldolase, class I [EC:4.1.2.13]
K01624	fructose-bisphosphate aldolase, class II [EC:4.1.2.13]
K01676	fumarate hydratase, class I [EC:4.2.1.2]
K01677	fumarate hydratase subunit alpha [EC:4.2.1.2]
K01678	fumarate hydratase subunit beta [EC:4.2.1.2]
K01679	fumarate hydratase, class II [EC:4.2.1.2]
K01681	aconitate hydratase [EC:4.2.1.3]
K01682	aconitate hydratase 2 / 2-methylisocitrate dehydratase [EC:4.2.1.3 4.2.1.99]
K01783	ribulose-phosphate 3-epimerase [EC:5.1.3.1]
K01803	triosephosphate isomerase (TIM) [EC:5.3.1.1]
K01807	ribose 5-phosphate isomerase A [EC:5.3.1.6]
K01808	ribose 5-phosphate isomerase B [EC:5.3.1.6]
K01847	methylmalonyl-CoA mutase [EC:5.4.99.2]
K01848	methylmalonyl-CoA mutase, N-terminal domain [EC:5.4.99.2]
K01849	methylmalonyl-CoA mutase, C-terminal domain [EC:5.4.99.2]
K01902	succinyl-CoA synthetase alpha subunit [EC:6.2.1.5]
K01903	succinyl-CoA synthetase beta subunit [EC:6.2.1.5]
K01907	acetoacetyl-CoA synthetase [EC:6.2.1.16]
K01938	formate--tetrahydrofolate ligase [EC:6.3.4.3]
K01958	pyruvate carboxylase [EC:6.4.1.1]
K01959	pyruvate carboxylase subunit A [EC:6.4.1.1]
K01960	pyruvate carboxylase subunit B [EC:6.4.1.1]
K01961	acetyl-CoA carboxylase, biotin carboxylase subunit [EC:6.4.1.2 6.3.4.14]
K01962	acetyl-CoA carboxylase carboxyl transferase subunit alpha [EC:6.4.1.2 2.1.3.15]
K01963	acetyl-CoA carboxylase carboxyl transferase subunit beta [EC:6.4.1.2 2.1.3.15]
K01964	acetyl-CoA/propionyl-CoA carboxylase [EC:6.4.1.2 6.4.1.3]
K02160	acetyl-CoA carboxylase biotin carboxyl carrier protein
K02446	fructose-1,6-bisphosphatase II [EC:3.1.3.11]
K03737	pyruvate-ferredoxin/flavodoxin oxidoreductase [EC:1.2.7.1 1.2.7.-]
K03841	fructose-1,6-bisphosphatase I [EC:3.1.3.11]
K05299	formate dehydrogenase (NADP+) alpha subunit [EC:1.17.1.10]
K05606	methylmalonyl-CoA/ethylmalonyl-CoA epimerase [EC:5.1.99.1]
K08691	malyl-CoA/(S)-citramalyl-CoA lyase [EC:4.1.3.24 4.1.3.25]
K09709	3-methylfumaryl-CoA hydratase [EC:4.2.1.153]
K11532	fructose-1,6-bisphosphatase II / sedoheptulose-1,7-bisphosphatase [EC:3.1.3.11 3.1.3.37]
K13788	phosphate acetyltransferase [EC:2.3.1.8]
K14138	acetyl-CoA synthase [EC:2.3.1.169]
K14449	2-methylfumaryl-CoA hydratase [EC:4.2.1.148]
K14465	succinate semialdehyde reductase (NADPH) [EC:1.1.1.-]
K14466	4-hydroxybutyrate---CoA ligase (AMP-forming) [EC:6.2.1.40]
K14467	4-hydroxybutyrate---CoA ligase (AMP-forming) [EC:6.2.1.40]
K14468	malonyl-CoA reductase / 3-hydroxypropionate dehydrogenase (NADP+) [EC:1.2.1.75 1.1.1.298]
K14469	acrylyl-CoA reductase (NADPH) / 3-hydroxypropionyl-CoA dehydratase / 3-hydroxypropionyl-CoA synthetase [EC:1.3.1.84 4.2.1.116 6.2.1.36]
K14470	2-methylfumaryl-CoA isomerase [EC:5.4.1.3]
K14471	succinyl-CoA:(S)-malate CoA-transferase subunit A [EC:2.8.3.22]
K14472	succinyl-CoA:(S)-malate CoA-transferase subunit B [EC:2.8.3.22]

	K14534	4-hydroxybutyryl-CoA dehydratase / vinylacetyl-CoA-Delta-isomerase [EC:4.2.1.120 5.3.3.3]
	K15016	enoyl-CoA hydratase / 3-hydroxyacyl-CoA dehydrogenase [EC:4.2.1.17 1.1.1.35]
	K15018	3-hydroxypropionyl-coenzyme A synthetase [EC:6.2.1.36]
	K15019	3-hydroxypropionyl-coenzyme A dehydratase [EC:4.2.1.116]
	K15020	acryloyl-coenzyme A reductase [EC:1.3.1.84]
	K15022	formate dehydrogenase (NADP+) beta subunit [EC:1.17.1.10]
	K15023	5-methyltetrahydrofolate corrinoid/iron sulfur protein methyltransferase [EC:2.1.1.258]
	K15024	putative phosphotransacetylase [EC:2.3.1.8]
	K15036	acetyl-CoA/propionyl-CoA carboxylase [EC:6.4.1.2 6.4.1.3 2.1.3.15]
	K15038	succinyl-CoA reductase [EC:1.2.1.76]
	K15039	3-hydroxypropionate dehydrogenase (NADP+) [EC:1.1.1.298]
	K15052	propionyl-CoA carboxylase [EC:6.4.1.3 2.1.3.15]
	K15230	ATP-citrate lyase alpha-subunit [EC:2.3.3.8]
	K15231	ATP-citrate lyase beta-subunit [EC:2.3.3.8]
	K15232	citryl-CoA synthetase large subunit [EC:6.2.1.18]
	K15234	citryl-CoA lyase [EC:4.1.3.34]
	K18209	fumarate reductase (CoM/CoB) subunit A [EC:1.3.4.1]
	K18210	fumarate reductase (CoM/CoB) subunit B [EC:1.3.4.1]
	K18556	NADH-dependent fumarate reductase subunit A [EC:1.3.1.6]
	K18560	NADH-dependent fumarate reductase subunit E
	K18594	3-hydroxypropionyl-CoA synthetase (ADP-forming) [EC:6.2.1.-]
	K18602	malonic semialdehyde reductase [EC:1.1.1.-]
	K18603	acetyl-CoA/propionyl-CoA carboxylase [EC:6.4.1.2 6.4.1.3]
	K18604	acetyl-CoA/propionyl-CoA carboxylase [EC:6.4.1.2 6.4.1.3 2.1.3.15]
	K18605	biotin carboxyl carrier protein
	K19280	succinyl-CoA:mesaconate CoA transferase [EC:2.8.3.26]
	K22015	formate dehydrogenase (acceptor) [EC:1.17.98.4 1.17.98.5]
Cellulose breakdown	K01179	endoglucanase [EC:3.2.1.4]
	K05349	beta-glucosidase [EC:3.2.1.21]
	K05350	beta-glucosidase [EC:3.2.1.21]
	K16213	cellobiose epimerase [EC:5.1.3.11]
	K19668	cellulose 1,4-beta-cellobiosidase [EC:3.2.1.91]
Hemicellulose breakdown	K01181	endo-1,4-beta-xylanase [EC:3.2.1.8]
	K01198	xylan 1,4-beta-xylosidase [EC:3.2.1.37]
	K01218	mannan endo-1,4-beta-mannosidase [EC:3.2.1.78]
	K01224	arabinogalactan endo-1,4-beta-galactosidase [EC:3.2.1.89]
	K01684	galactonate dehydratase [EC:4.2.1.6]
	K01710	dTDP-glucose 4,6-dehydratase [EC:4.2.1.46]
	K01805	xylose isomerase [EC:5.3.1.5]
	K01811	alpha-D-xyloside xylohydrolase [EC:3.2.1.177]
	K10543	D-xylose transport system substrate-binding protein
	K10544	D-xylose transport system permease protein
	K15921	arabinoxylan arabinofuranohydrolase [EC:3.2.1.55]
	K15924	glucuronoarabinoxylan endo-1,4-beta-xylanase [EC:3.2.1.136]
Lignin breakdown	K00446	catechol 2,3-dioxygenase [EC:1.13.11.2]
	K03381	catechol 1,2-dioxygenase [EC:1.13.11.1]
	K08689	biphenyl 2,3-dioxygenase subunit alpha [EC:1.14.12.18]
	K14582	cis-1,2-dihydro-1,2-dihydroxynaphthalene/dibenzothiophene dihydrodiol dehydrogenase [EC:1.3.1.29 1.3.1.60]
	K15750	biphenyl 2,3-dioxygenase subunit beta [EC:1.14.12.18]
Chitin breakdown	K01183	chitinase [EC:3.2.1.14]
	K01452	chitin deacetylase [EC:3.5.1.41]

	K03791	putative chitinase
	K03933	chitin-binding protein
	K13381	bifunctional chitinase/lysozyme [EC:3.2.1.14 3.2.1.17]
Starch breakdown	K00705	4-alpha-glucanotransferase [EC:2.4.1.25]
	K01187	alpha-glucosidase [EC:3.2.1.20]
	K01214	isoamylase [EC:3.2.1.68]
Pectin breakdown	K01184	polygalacturonase [EC:3.2.1.15]
	K01728	pectate lyase [EC:4.2.2.2]
Methanogenesis and methane metabolism	K00018	glycerate dehydrogenase [EC:1.1.1.29]
	K00024	malate dehydrogenase [EC:1.1.1.37]
	K00125	formate dehydrogenase (coenzyme F420) beta subunit [EC:1.17.98.3 1.8.98.6]
	K00192	anaerobic carbon-monoxide dehydrogenase, complex subunit alpha [EC:1.2.7.4]
	K00193	acetyl-CoA decarbonylase/synthase, complex subunit beta [EC:2.3.1.169]
	K00194	acetyl-CoA decarbonylase/synthase, complex subunit delta [EC:2.1.1.245]
	K00195	acetyl-CoA decarbonylase/synthase complex subunit epsilon
	K00197	acetyl-CoA decarbonylase/synthase, complex subunit gamma [EC:2.1.1.245]
	K00200	formylmethanofuran dehydrogenase subunit A [EC:1.2.7.12]
	K00201	formylmethanofuran dehydrogenase subunit B [EC:1.2.7.12]
	K00202	formylmethanofuran dehydrogenase subunit C [EC:1.2.7.12]
	K00203	formylmethanofuran dehydrogenase subunit D [EC:1.2.7.12]
	K00204	4Fe-4S ferredoxin
	K00205	4Fe-4S ferredoxin
	K00319	methylenetetrahydromethanopterin dehydrogenase [EC:1.5.98.1]
	K00320	5,10-methylenetetrahydromethanopterin reductase [EC:1.5.98.2]
	K00399	methyl-coenzyme M reductase alpha subunit [EC:2.8.4.1]
	K00400	methyl coenzyme M reductase system, component A2
	K00401	methyl-coenzyme M reductase beta subunit [EC:2.8.4.1]
	K00402	methyl-coenzyme M reductase gamma subunit [EC:2.8.4.1]
	K00577	tetrahydromethanopterin S-methyltransferase subunit A [EC:7.2.1.4]
	K00578	tetrahydromethanopterin S-methyltransferase subunit B [EC:7.2.1.4]
	K00579	tetrahydromethanopterin S-methyltransferase subunit C [EC:7.2.1.4]
	K00580	tetrahydromethanopterin S-methyltransferase subunit D [EC:7.2.1.4]
	K00581	tetrahydromethanopterin S-methyltransferase subunit E [EC:7.2.1.4]
	K00582	tetrahydromethanopterin S-methyltransferase subunit F [EC:7.2.1.4]
	K00583	tetrahydromethanopterin S-methyltransferase subunit G [EC:7.2.1.4]
	K00584	tetrahydromethanopterin S-methyltransferase subunit H [EC:7.2.1.4]
	K00600	glycine hydroxymethyltransferase [EC:2.1.2.1]
	K00625	phosphate acetyltransferase [EC:2.3.1.8]
	K00672	formylmethanofuran--tetrahydromethanopterin N-formyltransferase [EC:2.3.1.101]
	K00830	alanine-glyoxylate transaminase / serine-glyoxylate transaminase / serine-pyruvate transaminase [EC:2.6.1.44 2.6.1.45 2.6.1.51]
	K00850	6-phosphofructokinase 1 [EC:2.7.1.11]
	K00863	triose/dihydroxyacetone kinase / FAD-AMP lyase (cyclizing) [EC:2.7.1.28 2.7.1.29 4.6.1.15]
	K00925	acetate kinase [EC:2.7.2.1]

K01499	methenyltetrahydromethanopterin cyclohydrolase [EC:3.5.4.27]
K01595	phosphoenolpyruvate carboxylase [EC:4.1.1.31]
K01624	fructose-bisphosphate aldolase, class II [EC:4.1.2.13]
K01689	enolase [EC:4.2.1.11]
K01895	acetyl-CoA synthetase [EC:6.2.1.1]
K03388	heterodisulfide reductase subunit A2 [EC:1.8.7.3 1.8.98.4 1.8.98.5 1.8.98.6]
K03389	heterodisulfide reductase subunit B2 [EC:1.8.7.3 1.8.98.4 1.8.98.5 1.8.98.6]
K03390	heterodisulfide reductase subunit C2 [EC:1.8.7.3 1.8.98.4 1.8.98.5 1.8.98.6]
K03841	fructose-1,6-bisphosphatase I [EC:3.1.3.11]
K04480	methanol---5-hydroxybenzimidazolylcobamide Co-methyltransferase [EC:2.1.1.90]
K08093	3-hexulose-6-phosphate synthase [EC:4.1.2.43]
K08094	6-phospho-3-hexuloisomerase [EC:5.3.1.27]
K08264	heterodisulfide reductase subunit D [EC:1.8.98.1]
K08265	heterodisulfide reductase subunit E [EC:1.8.98.1]
K08691	malyl-CoA/(S)-citramalyl-CoA lyase [EC:4.1.3.24 4.1.3.25]
K08692	malate-CoA ligase subunit alpha [EC:6.2.1.9]
K10944	methane/ammonia monooxygenase subunit A [EC:1.14.18.3 1.14.99.39]
K10945	methane/ammonia monooxygenase subunit B
K10946	methane/ammonia monooxygenase subunit C
K11260	4Fe-4S ferredoxin
K11261	formylmethanofuran dehydrogenase subunit E [EC:1.2.7.12]
K11529	glycerate 2-kinase [EC:2.7.1.165]
K13812	bifunctional enzyme Fae/Hps [EC:4.2.1.147 4.1.2.43]
K13831	3-hexulose-6-phosphate synthase / 6-phospho-3-hexuloisomerase [EC:4.1.2.43 5.3.1.27]
K14028	methanol dehydrogenase (cytochrome c) subunit 1 [EC:1.1.2.7]
K14029	methanol dehydrogenase (cytochrome c) subunit 2 [EC:1.1.2.7]
K14067	malate-CoA ligase subunit beta [EC:6.2.1.9]
K14080	[methyl-Co(III) methanol/glycine betaine-specific corrinoid protein]:coenzyme M methyltransferase [EC:2.1.1.246 2.1.1.377]
K14081	methanol corrinoid protein
K14082	[methyl-Co(III) methylamine-specific corrinoid protein]:coenzyme M methyltransferase [EC:2.1.1.247]
K14083	trimethylamine---corrinoid protein Co-methyltransferase [EC:2.1.1.250]
K14084	trimethylamine corrinoid protein
K14126	F420-non-reducing hydrogenase large subunit [EC:1.12.99.- 1.8.98.5]
K14127	F420-non-reducing hydrogenase iron-sulfur subunit [EC:1.12.99.- 1.8.98.5 1.8.98.6]
K14128	F420-non-reducing hydrogenase small subunit [EC:1.12.99.- 1.8.98.5]
K16157	methane monooxygenase component A alpha chain [EC:1.14.13.25]
K16158	methane monooxygenase component A beta chain [EC:1.14.13.25]
K16159	methane monooxygenase component A gamma chain [EC:1.14.13.25]
K16160	methane monooxygenase regulatory protein B
K16161	methane monooxygenase component C [EC:1.14.13.25]
K16162	methane monooxygenase component D
K16176	methylamine---corrinoid protein Co-methyltransferase [EC:2.1.1.248]
K16177	monomethylamine corrinoid protein
K16178	dimethylamine---corrinoid protein Co-methyltransferase

			[EC:2.1.1.249]
		K16179	dimethylamine corrinoid protein
		K16370	6-phosphofructokinase 2 [EC:2.7.1.11]
		K21071	ATP-dependent phosphofructokinase / diphosphate-dependent phosphofructokinase [EC:2.7.1.11 2.7.1.90]
		K22480	heterodisulfide reductase subunit A1 [EC:1.8.7.3]
		K22481	heterodisulfide reductase subunit B1 [EC:1.8.7.3]
		K22482	heterodisulfide reductase subunit C1 [EC:1.8.7.3]
		K22516	formate dehydrogenase (coenzyme F420) alpha subunit [EC:1.17.98.3 1.8.98.6]
	Nitrification	K00370	nitrate reductase / nitrite oxidoreductase, alpha subunit [EC:1.7.5.1 1.7.99.-]
		K00371	nitrate reductase / nitrite oxidoreductase, beta subunit [EC:1.7.5.1 1.7.99.-]
		K10535	hydroxylamine dehydrogenase [EC:1.7.2.6]
		K10944	methane/ammonia monooxygenase subunit A [EC:1.14.18.3 1.14.99.39]
		K10945	methane/ammonia monooxygenase subunit B
		K10946	methane/ammonia monooxygenase subunit C
	Denitrification	K00368	nitrite reductase (NO-forming) [EC:1.7.2.1]
		K00370	nitrate reductase / nitrite oxidoreductase, alpha subunit [EC:1.7.5.1 1.7.99.-]
		K00371	nitrate reductase / nitrite oxidoreductase, beta subunit [EC:1.7.5.1 1.7.99.-]
		K00373	nitrate reductase molybdenum cofactor assembly chaperone NarJ/NarW
		K00374	nitrate reductase gamma subunit [EC:1.7.5.1 1.7.99.-]
		K00376	nitrous-oxide reductase [EC:1.7.2.4]
		K02305	nitric oxide reductase subunit C
		K02567	periplasmic nitrate reductase NapA [EC:1.7.99.-]
		K02568	cytochrome c-type protein NapB
		K02569	cytochrome c-type protein NapC
		K04561	nitric oxide reductase subunit B [EC:1.7.2.5]
		K15864	nitrite reductase (NO-forming) / hydroxylamine reductase [EC:1.7.2.1 1.7.99.1]
Nitrogen	Assimilatory nitrate reduction	K00265	glutamate synthase (NADPH/NADH) large chain [EC:1.4.1.13 1.4.1.14]
		K00284	glutamate synthase (ferredoxin) [EC:1.4.7.1]
		K00360	assimilatory nitrate reductase electron transfer subunit [EC:1.7.99.-]
		K00366	ferredoxin-nitrite reductase [EC:1.7.7.1]
		K00367	ferredoxin-nitrate reductase [EC:1.7.7.2]
		K00372	assimilatory nitrate reductase catalytic subunit [EC:1.7.99.-]
		K01915	glutamine synthetase [EC:6.3.1.2]
		K02575	MFS transporter, NNP family, nitrate/nitrite transporter
		K15576	nitrate/nitrite transport system substrate-binding protein
		K15577	nitrate/nitrite transport system permease protein
		K15578	nitrate/nitrite transport system ATP-binding protein [EC:3.6.3.-]
		K15579	nitrate/nitrite transport system ATP-binding protein
		K15879	cytochrome b-561
	Dissimilatory nitrate reduction	K00362	nitrite reductase (NADH) large subunit [EC:1.7.1.15]
		K00363	nitrite reductase (NADH) small subunit [EC:1.7.1.15]
		K00370	nitrate reductase / nitrite oxidoreductase, alpha subunit [EC:1.7.5.1 1.7.99.-]
		K00371	nitrate reductase / nitrite oxidoreductase, beta subunit [EC:1.7.5.1 1.7.99.-]
		K00373	nitrate reductase molybdenum cofactor assembly chaperone NarJ/NarW
		K00374	nitrate reductase gamma subunit [EC:1.7.5.1 1.7.99.-]
		K02567	periplasmic nitrate reductase NapA [EC:1.7.99.-]
		K02568	cytochrome c-type protein NapB
		K02569	cytochrome c-type protein NapC
		K03385	nitrite reductase (cytochrome c-552) [EC:1.7.2.2]

		K04013	cytochrome c-type protein NrfB
		K04014	protein NrfC
		K04015	protein NrfD
		K15876	cytochrome c nitrite reductase small subunit
	Nitrogen fixation	K00531	nitrogenase delta subunit [EC:1.18.6.1]
		K02586	nitrogenase molybdenum-iron protein alpha chain [EC:1.18.6.1]
		K02588	nitrogenase iron protein NifH [EC:1.18.6.1]
		K02591	nitrogenase molybdenum-iron protein beta chain [EC:1.18.6.1]
		K02592	nitrogenase molybdenum-iron protein NifN
		K02593	nitrogen fixation protein NifT
		K02594	homocitrate synthase NifV [EC:2.3.3.14]
		K02595	nitrogenase-stabilizing/protective protein
		K02596	nitrogen fixation protein NifX
		K02597	nitrogen fixation protein NifZ
	Anammox	K00368	nitrite reductase (NO-forming) [EC:1.7.2.1]
		K01428	urease subunit alpha [EC:3.5.1.5]
		K01429	urease subunit beta [EC:3.5.1.5]
		K01430	urease subunit gamma [EC:3.5.1.5]
		K10535	hydroxylamine dehydrogenase [EC:1.7.2.6]
		K15864	nitrite reductase (NO-forming) / hydroxylamine reductase [EC:1.7.2.1 1.7.99.1]
	Nitrogen mineralization	K00260	glutamate dehydrogenase [EC:1.4.1.2]
		K00261	glutamate dehydrogenase (NAD(P)+) [EC:1.4.1.3]
		K00262	glutamate dehydrogenase (NADP+) [EC:1.4.1.4]
	Nitrogen assimilation	K00265	glutamate synthase (NADPH/NADH) large chain [EC:1.4.1.13 1.4.1.14]
		K00266	glutamate synthase (NADPH/NADH) small chain [EC:1.4.1.13 1.4.1.14]
		K00284	glutamate synthase (ferredoxin) [EC:1.4.7.1]
		K00459	nitronate monooxygenase [EC:1.13.12.16]
		K01424	L-asparaginase [EC:3.5.1.1]
		K01425	glutaminase [EC:3.5.1.2]
		K01915	glutamine synthetase [EC:6.3.1.2]
		K01953	asparagine synthase (glutamine-hydrolysing) [EC:6.3.5.4]
Phosphorus	Polyphosphate consolidation	K00937	polyphosphate kinase [EC:2.7.4.1]
		K15986	manganese-dependent inorganic pyrophosphatase [EC:3.6.1.1]
	Polyphosphate degradation	K00858	NAD+ kinase [EC:2.7.1.23]
		K00873	pyruvate kinase [EC:2.7.1.40]
		K00886	polyphosphate glucokinase [EC:2.7.1.63]
		K00940	nucleoside-diphosphate kinase [EC:2.7.4.6]
		K00951	GTP pyrophosphokinase [EC:2.7.6.5]
		K01139	GTP diphosphokinase / guanosine-3,5-bis(diphosphate) 3-diphosphatase [EC:2.7.6.5 3.1.7.2]
		K03787	5-nucleotidase [EC:3.1.3.5]
		K22468	polyphosphate kinase [EC:2.7.4.1]
	P-starvation responseregulation	K02039	phosphate transport system protein
		K07636	two-component system, OmpR family, phosphate regulon sensor histidine kinase PhoR [EC:2.7.13.3]
		K07657	two-component system, OmpR family, phosphate regulon response regulator PhoB
		K07658	two-component system, OmpR family, alkaline phosphatase synthesis response regulator PhoP
		K10916	two-component system, CAI-1 autoinducer sensor kinase/phosphatase CqsS [EC:2.7.13.3 3.1.3.-]
	P-uptake and transport system	K00937	polyphosphate kinase [EC:2.7.4.1]
		K02036	phosphate transport system ATP-binding protein [EC:3.6.3.27]
		K02037	phosphate transport system permease protein
		K02038	phosphate transport system permease protein
		K02040	phosphate transport system substrate-binding protein

	K02041	phosphonate transport system ATP-binding protein [EC:3.6.3.28]
	K02042	phosphonate transport system permease protein
	K02043	GntR family transcriptional regulator, phosphonate transport system regulatory protein
	K02044	phosphonate transport system substrate-binding protein
	K02433	aspartyl-tRNA(Asn)/glutamyl-tRNA(Gln) amidotransferase subunit A [EC:6.3.5.6 6.3.5.7]
	K02440	glycerol uptake facilitator protein
	K02443	glycerol uptake operon antiterminator
	K02444	DeoR family transcriptional regulator, glycerol-3-phosphate regulon repressor
	K02445	MFS transporter, OPA family, glycerol-3-phosphate transporter
	K02757	PTS system, beta-glucoside-specific IIC component
	K03306	inorganic phosphate transporter, PiT family
	K03324	phosphate:Na+ symporter
	K05781	putative phosphonate transport system ATP-binding protein
	K05813	sn-glycerol 3-phosphate transport system substrate-binding protein
	K05814	sn-glycerol 3-phosphate transport system permease protein
	K05815	sn-glycerol 3-phosphate transport system permease protein
	K05816	sn-glycerol 3-phosphate transport system ATP-binding protein [EC:3.6.3.20]
	K05833	putative ABC transport system ATP-binding protein
	K07220	uncharacterized protein
	K07221	phosphate-selective porin OprO and OprP
	K16322	low-affinity inorganic phosphate transporter
Inorganic P-solubilization	K00112	glycerol-3-phosphate dehydrogenase subunit B [EC:1.1.5.3]
	K00113	glycerol-3-phosphate dehydrogenase subunit C [EC:1.1.5.3]
	K00117	quinoprotein glucose dehydrogenase [EC:1.1.5.2]
	K00937	polyphosphate kinase [EC:2.7.4.1]
	K01507	inorganic pyrophosphatase [EC:3.6.1.1]
	K01524	exopolyphosphatase / guanosine-5-triphosphate,3-diphosphate pyrophosphatase [EC:3.6.1.11 3.6.1.40]
	K06136	pyrroloquinoline quinone biosynthesis protein B
	K06137	pyrroloquinoline-quinone synthase [EC:1.3.3.11]
	K06138	pyrroloquinoline quinone biosynthesis protein D
	K06139	pyrroloquinoline quinone biosynthesis protein E
Organic P-mineralization	K00105	alpha-glycerophosphate oxidase [EC:1.1.3.21]
	K00111	glycerol-3-phosphate dehydrogenase [EC: 1.1.5.3]
	K00112	glycerol-3-phosphate dehydrogenase subunit B [EC:1.1.5.3]
	K00113	glycerol-3-phosphate dehydrogenase subunit C [EC:1.1.5.3]
	K00864	glycerol kinase [EC:2.7.1.30]
	K00906	isocitrate dehydrogenase kinase/phosphatase [EC:2.7.11.5 3.1.3.-]
	K01077	alkaline phosphatase [EC:3.1.3.1]
	K01079	phosphoserine phosphatase [EC:3.1.3.3]
	K01083	3-phytase [EC:3.1.3.8]
	K01091	phosphoglycolate phosphatase [EC:3.1.3.18]
	K01092	myo-inositol-1(or 4)-monophosphatase [EC:3.1.3.25]
	K01093	4-phytase / acid phosphatase [EC:3.1.3.26 3.1.3.2]
	K01113	alkaline phosphatase D [EC:3.1.3.1]
	K01126	glycerophosphoryl diester phosphodiesterase [EC:3.1.4.46]
	K01841	phosphoenolpyruvate phosphomutase [EC:5.4.2.9]
	K02043	GntR family transcriptional regulator, phosphonate transport system regulatory protein
	K02203	phosphoserine / homoserine phosphotransferase [EC:3.1.3.3 2.7.1.39]
	K02440	glycerol uptake facilitator protein
	K02444	DeoR family transcriptional regulator, glycerol-3-phosphate regulon repressor

K03270	3-deoxy-D-manno-octulosonate 8-phosphate phosphatase (KDO 8-P phosphatase) [EC:3.1.3.45]
K03430	2-aminoethylphosphonate-pyruvate transaminase [EC:2.6.1.37]
K03788	acid phosphatase (class B) [EC:3.1.3.2]
K05306	phosphonoacetaldehyde hydrolase [EC:3.11.1.1]
K05518	phosphoserine phosphatase RsbX [EC:3.1.3.3]
K05774	ribose 1,5-bisphosphokinase [EC:2.7.4.23]
K05780	alpha-D-ribose 1-methylphosphonate 5-triphosphate synthase subunit PhnL [EC:2.7.8.37]
K05781	putative phosphonate transport system ATP-binding protein
K06162	alpha-D-ribose 1-methylphosphonate 5-triphosphate diphosphatase [EC:3.6.1.63]
K06163	alpha-D-ribose 1-methylphosphonate 5-phosphate C-P lyase [EC:4.7.1.1]
K06164	alpha-D-ribose 1-methylphosphonate 5-triphosphate synthase subunit PhnI [EC:2.7.8.37]
K06165	alpha-D-ribose 1-methylphosphonate 5-triphosphate synthase subunit PhnH [EC:2.7.8.37]
K06166	alpha-D-ribose 1-methylphosphonate 5-triphosphate synthase subunit PhnG [EC:2.7.8.37]
K06167	phosphoribosyl 1,2-cyclic phosphate phosphodiesterase [EC:3.1.4.55]
K06193	protein PhnA
K07048	phosphotriesterase-related protein
K07175	PhoH-like ATPase
K07315	phosphoserine phosphatase RsbU/P [EC:3.1.3.3]
K08483	phosphotransferase system, enzyme I, PtsI [EC:2.7.3.9]
K08484	phosphotransferase system, enzyme I, PtsP [EC:2.7.3.9]
K09474	acid phosphatase (class A) [EC:3.1.3.2]
K09994	aminoalkylphosphonate N-acetyltransferase [EC:2.3.1.-]
K15781	putative phosphoserine phosphatase / 1-acylglycerol-3-phosphate O-acyltransferase [EC:3.1.3.3 2.3.1.51]
K16055	trehalose 6-phosphate synthase/phosphatase [EC:2.4.1.15 3.1.3.12]

The classification and annotation results of soil carbon, nitrogen, and phosphorus cycling functional genes refer to Bai, Du, Liu, Xiao, and the KEGG database [1–4].

References

1. Bai, C.H. Effects of shrub presence on soil microbial functional genes abundance for carbon, nitrogen and phosphorus cyclings in desert ecosystem. Master, Xinjiang Agricultural University, 2023. DOI:10.27431/d.cnki.gxnyu.2023.000373.
2. Du, L.; Zhong, H.H.; Guo, X.N.; Li, H.N.; Xia, J.X.; Chen, Q. Nitrogen fertilization and soil nitrogen cycling: Unraveling the links among multiple environmental factors, functional genes, and transformation rates. *Sci. Total Environ.* **2024**, 951175561. https://doi.org/10.1016/j.scitotenv.2024.175561.
3. Liu, L. Response characteristics and mechanism of soil microbial community and function to long-term phosphorus fertilizer input in dryland wheat cropping system. Doctorate, Northwest Agriculture and Forestry University, 2024. DOI:10.27409/d.cnki.gxbnu.2024.002915.
4. Xiao, Z.R. Effects of coniferous plantation conversion on soil phosphorus cyclin microbial functional genes and species in south subtropical China. Master, Guangxi University, 2024. DOI:10.27034/d.cnki.ggxiu.2024.000204.
